# Novel Clade 2.3.4.4b Highly Pathogenic Avian Influenza A H5N8 and H5N5 Viruses in Denmark, 2020

**DOI:** 10.3390/v13050886

**Published:** 2021-05-11

**Authors:** Yuan Liang, Jakob N. Nissen, Jesper S. Krog, Solvej Ø. Breum, Ramona Trebbien, Lars E. Larsen, Charlotte K. Hjulsager

**Affiliations:** 1 Department of Veterinary and Animal Sciences, University of Copenhagen, 1870 Frederiksberg, Denmark; yuan.liang@sund.ku.dk (Y.L.); lael@sund.ku.dk (L.E.L.); 2 Virus and Microbiological Special Diagnostics, Statens Serum Institut, 2300 Copenhagen S, Denmark; JANN@ssi.dk (J.N.N.); JSKR@ssi.dk (J.S.K.); SOBR@ssi.dk (S.Ø.B.); RATR@ssi.dk (R.T.)

**Keywords:** influenza A virus, H5N8 subtype, influenza A virus, H5N5 subtype, disease outbreaks, influenza in birds, phylogeny, Europe, poultry, wild

## Abstract

Since late 2020, outbreaks of H5 highly pathogenic avian influenza (HPAI) viruses belonging to clade 2.3.4.4b have emerged in Europe. To investigate the evolutionary history of these viruses, we performed genetic characterization on the first HPAI viruses found in Denmark during the autumn of 2020. H5N8 viruses from 14 wild birds and poultry, as well as one H5N5 virus from a wild bird, were characterized by whole genome sequencing and phylogenetic analysis. The Danish H5N8 viruses were found to be genetically similar to each other and to contemporary European clade 2.3.4.4b H5N8 viruses, while the Danish H5N5 virus was shown to be a unique genotype from the H5N5 viruses that circulated at the same time in Russia, Germany, and Belgium. Genetic analyses of one of the H5N8 viruses revealed the presence of a substitution (PB2-M64T) that is highly conserved in human seasonal influenza A viruses. Our analyses showed that the late 2020 clade 2.3.4.4b HPAI H5N8 viruses were most likely new incursions introduced by migrating birds to overwintering sites in Europe, rather than the result of continued circulation of H5N8 viruses from previous introductions to Europe in 2016/2017 and early 2020.

## 1. Introduction

Avian influenza viruses (AIV) are divided into low pathogenic AI (LPAI) and highly pathogenic AI (HPAI) viruses based on their pathogenicity in chickens. Incursions of HPAI H5NX viruses belonging to clade 2.3.4.4 of the A/Goose/Guangdong/1/96 (Gs/Gd) lineage have caused outbreaks in Europe on several occasions since 2014 [1,2]. The 2016/2017 epidemic was especially severe and caused substantial losses among wild water birds and commercial poultry across Europe [1]. In Denmark, outbreaks of HPAI clade 2.3.4.4b H5N8 in wild birds and two backyard poultry flocks were observed in the winter of 2016/2017 [3], while HPAI clade 2.3.4.4b H5N6 affected wild birds in 2018 [4]. Starting in late 2019 and lasting until June 2020, novel HPAI clade 2.3.4.4b H5N8 viruses caused outbreaks in Central and Eastern European countries. These viruses were generated by reassortment between H5N8 from sub-Saharan Africa and LPAI viruses from Eurasia [5,6]. Since October 2020, clade 2.3.4.4b HPAI H5 viruses have been detected again in both wild birds and poultry in Europe, initially in The Netherlands, Germany, and Denmark, then shortly after in many other European countries [7,8]. Most viruses were H5N8, but related H5N5 reassortant viruses have been reported since late 2020. Here, we report the genetic characterization of the first HPAI clade 2.3.4.4b H5N8 and H5N5 viruses detected in wild birds and poultry in Denmark during the autumn of 2020.

## 2. Materials and Methods

### 2.1. Viruses

The study included the first HPAI viruses detected in Denmark in 2020 and consisted of 14 HPAI H5N8 viruses and one HPAI H5N5 virus detected as part of the EU mandatory passive surveillance for AIV in wild birds found dead in the environment. In addition, an HPAI H5N8 virus from the first outbreak of HPAI in poultry in Denmark in 2020 was included. Suspicion of AIV was raised on 15 November 2020, due to mortality, diarrhea, vomiting, lethargy, and a decrease in egg production and feed and water intake. The flock consisted of 25,000 layers that produced hatching eggs for broiler production.

### 2.2. Full Genome Sequencing

RNA was extracted with RNeasy Mini Kit (QIAGEN, Copenhagen, Denmark) from 200 µL swab material mixed with 400 µL Buffer RLT with 1% beta-mercaptoethanol. RNA extraction was automated on the QIAcube extraction robot (QIAGEN QIAcube, RRID:SCR_020414) using the large sample protocol version 2 and 100 µL elution volume.

Next-generation sequencing was performed on one-tube eight-segment mixtures amplified in a single tube from each sample using the primers, MBTuni12R and MBTuni13 [9] and SuperScript III One-Step RT-PCR System with Platinum Taq High Fidelity kit (Invitrogen, Thermo Fischer Scientific, Roskilde, Denmark) on a T3 thermocycler (Biometra, Nærum, Denmark) under the following conditions: 42 °C for 60 min; 5 cycles of 94 °C for 30 s, 45 °C for 30 s, 68 °C for 180 s; 31 cycles of 94 °C for 30 s, 57 °C for 30 s, 68 °C for 180 s; and 7 min at 68 °C. The amplified mixtures were purified with Illustra GFX PCR DNA and a Gel Band Purification kit (GE Healthcare, Brøndby, Denmark). Libraries were built with the Nextera XT DNA Library Preparation Kit and sequenced on an Illumina MiSeq system (Illumina MiSeq System, RRID:SCR_016379) (Illumina, Copenhagen, Denmark) with MiSeq Reagent Kit v2, 50 cycles, according to instructions from the supplier.

EPI_ISL_644737 and EPI_ISL_644824 consensus sequences were generated with CLC Genomics Workbench version 20.0.4 (QIAGEN, RRID:SCR_011853) (QIAGEN, Aarhus, Denmark) using the default settings and paired reads. First, reads were de novo assembled, and matching reference sequences to the de novo assembled contigs were selected using Basic Local Alignment Search Tool (BLAST) similarity searches (NCBI BLAST, RRID:SCR_004870) in GenBank (NCBI, GenBank, RRID:SCR_002760) and EpiFlu^TM^ (GISAID, Global Initiative on Sharing All Influenza Data, RRID:SCR_018251) databases. Next, reads were mapped to the selected reference sequences, consensus sequences were extracted, and uni12R and Uni13 PCR primer binding regions were removed. For the remaining viruses, fastq sequence data files were initially trimmed with AdapterRemoval version 2.3.1 (AdapterRemoval, RRID:SCR_011834) [10] and quality controlled with FastQC version 0.11.9 (FastQC, RRID:SCR_014583) [11]. To find the most suitable reference sequences, we first downloaded all published H5 AIV genomes with all eight segments available in the GISAID database from 1 January 2020 to 30 November 2020. Then, for each segment/sample combination, we found the best reference sequence by mapping the sample’s reads to the references using sparse mapping on KMA version 1.3.8 [12] and reconstructed the consensus sequence using KMA regular alignment with the Mt1 option. In silico translation, sequence comparisons, and all further sequence processing was completed manually using BioSequences version 2.0.5.

### 2.3. Phylogenetic Analysis

Consensus sequences of each segment were aligned using MAFFT version 7.471 (MAFFT, RRID:SCR_011811) [13] with the default options. Maximum likelihood trees were generated from the alignment using IQ-TREE version 2.0.3 (IQ-TREE, RRID:SCR_017254) [14] with parameters “-bb 1000-nm 2500” and the model HKY+G2.

For the molecular dating analysis, model testing was performed for each gene segment with CLC Genomics Workbench version 8.0.2 (CLC Genomics Workbench, RRID:SCR_011853) (QIAGEN, Aarhus, Denmark). A neighbor-joining tree with 1000 bootstrap replicates was constructed using the same software to check for the presence of temporal signal by TempEST (TempEST, RRID:SCR_017304) [15]. Molecular clock trees were constructed using BEAST2 version 2.5.2 (BEAST, RRID:SCR_010228) [16]. All analyses were performed with gamma distributed rates over sites, a strict molecular clock model, and a birth–death skyline serial model. The prior for the reproduction number was set to log normal, with a lower and upper value of 0 and 10, respectively, and with M = 0.0, S = 1.0 and 5 dimensions. The prior for “becomeUninfectiousRate” was log normal with M = 52.0 and Y = 1.0, corresponding to an average of 52 per year; i.e., an average time of being infectious near 1 week. The prior for the sampling proportion was set to log normal with M = 0.001 and S = 1.25. The clock rate was set to log normal with M = 0.001 and S = 1.25. The prior for the origin was set to gamma with alpha = 0.5 and beta = 2.0. The remaining priors were set at default values. MCMC was run for 100,000,000 iterations with pre-burn-in set to 10%. Maximum clade credibility trees were generated with TreeAnnotator, with burn-in set to 50% and the posterior limit set at 0.5. The resulting trees were visualized using FigTree version 1.4.4 (FigTree, RRID:SCR_008515) [17]. Each analysis was run twice. Tracer version 1.7.1 (Tracer, RRID:SCR_019121) [18] was used to check the convergence.

### 2.4. Code Availability

All code and environment files are available at https://github.com/jakobnissen/2020hpai.

## 3. Results

**Figure 1:**
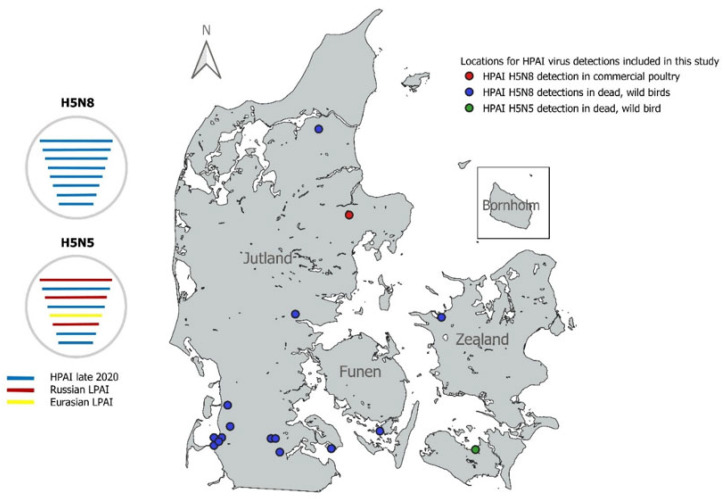
Schematic representation of the HPAI H5N8 and H5N5 viruses and geographic distribution of the viruses included in this study. The schematic representation shows the likely origin of the individual gene segments of the Danish H5N8 and H5N5 viruses. Blue bars represent gene segments that were most closely related to European HPAI H5N8 viruses from late 2020. Red bars represent segments that were closely related to Russian LPAI viruses from wild birds. Yellow bars represent gene segments that were closely related to LPAI viruses from wild birds elsewhere in Eurasia. On the map, every dot represents one bird found infected with HPAI virus. The blue dots represent dead, wild birds infected with HPAI H5N8 viruses. The red dot denotes the HPAI H5N8 detection from a commercial poultry. The green dot represents the H5N5 detection in a dead, wild peregrine falcon. The map was constructed with QGIS version 13.18.2 [20] using an outline of Denmark from Kortforsyningen (www.kortforsyningen.dk, downloaded 26 April 2012).

The first detection of clade 2.3.4.4b HPAI H5 virus in Denmark in 2020 was in a wild peregrine falcon (*Falco peregrinus*) found dead in Guldborgsund, Denmark, on 30 October 2020 (Figure 1). The virus detected in this bird was the HPAI H5N5 subtype. After this first detection, there were several further detections of HPAI H5N8 viruses in dead wild birds in Denmark during the remaining months of 2020 [19]. In this study, we included HPAI H5N8 viruses detected in nine Barnacle geese (*Branta leucopsis*), a black-headed gull (*Chroicocephalus ridibundus*), a graylag goose (*Anser anser*), a peregrine falcon (*Falco peregrinus*), and a common buzzard (*Buteo buteo*) that were all found dead between 3 and 9 November 2020 at various locations. One HPAI H5N8 virus detected in a commercial poultry farm in Tustrup, Randers, was also included in this analysis. Detailed information on these 15 H5N8 and the single H5N5 viruses is presented in Supplementary Table S1.

The H5N8 poultry virus did not differ genetically from the wild bird viruses. For all eight gene segments, the 15 Danish H5N8 viruses shared nucleotide sequence identities ranging from 99.0–100%. Phylogenetic analyses performed on all gene segments (Supplementary Figure S1) revealed that the H5N8 viruses belonged to HPAI H5 clade 2.3.4.4b, and all eight gene segments shared a close relatedness to contemporary European HPAI H5N8 viruses. These late 2020 H5N8 viruses were most closely related to A/chicken/Iraq/1/2020 in all gene segments and are referred to as “Iraqi-like” viruses. Thus, the Danish HPAI H5N8 viruses were genetically distinct from the European H5N8 viruses detected in Germany, Poland, Czech Republic, and Hungary in the first semester of 2020 and in Europe 2016/2017 (Supplementary Figure S1). Phylogenetic analyses were also performed on all gene segments of the peregrine falcon HPAI H5N5 virus. These analyses showed that this virus contained polymerase basic protein 1 (PB1), hemagglutinin (HA), matrix protein (MP), and nonstructural protein (NS) gene segments that were most closely related to the contemporary “Iraqi-like” European HPAI H5N8 viruses from the autumn of 2020 (Supplementary Figure S1), whereas the polymerase basic protein 2 (PB2), polymerase acidic protein (PA), and neuraminidase (NA) gene segments were closely related to Russian LPAI viruses from 2018, and the NP segment was most closely related to HPAI A/mallard duck/Korea/WA137/2017(H5N8) and LPAI viruses from Europe. In contrast, Russian HPAI H5N5 viruses from October 2020 contained a related NA segment, but the remaining segments were similar to “Iraqi-like” European HPAI H5N8 viruses from late 2020. H5N5 viruses detected in Germany and Belgium in 2020 also contained the “Iraqi-like” HPAI H5N8 backbone, but their PA and NA gene segments were most related to Russian LPAI viruses (Supplementary Figure S1). Thus, the Danish peregrine falcon H5N5 virus gene constellation was unique by having PB2, PA, NP, and NA segments most closely related to Eurasian/Russian LPAI virus.

**Figure 2:**
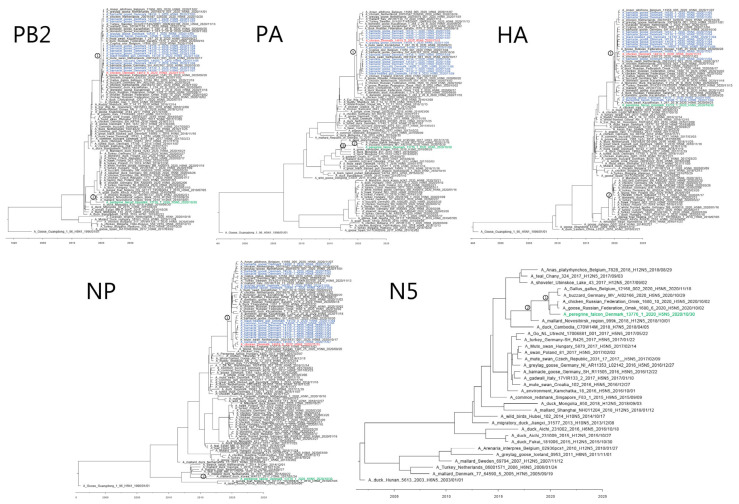
Maximum clade credibility trees of PB2, PA, HA, NP, and N5 gene segments from strict molecular clock analyses. The trees include gene segment sequences from Danish H5N8 and H5N5 viruses from 2020, representative Eurasian clade 2.3.4.4 HPAI H5 viruses and LPAI viruses. The numbers above the nodes represent time to most recent common ancestor (tMRCA). Tip labels are colored according to the background of the detections. Green: H5N5 wild bird virus; red: H5N8 virus from poultry; blue: H5N8 viruses from wild birds. The scale bar depicts the timeline. PB2, polymerase basic protein 2; PA, polymerase acidic protein; HA, hemagglutinin; NP, nucleoprotein; NA, neuraminidase.

The time to most recent common ancestor (tMRCA) was calculated to explain the emergence of the H5N8 and H5N5 viruses. Taking the intersection of the 95% highest posterior density (HPD) intervals of the tMRCA, we estimated the ancestral virus to have emerged around November 2019 (Figure 2, all gene segments, node 1) (Table 1, all gene segments, node 1) (Supplementary Figure S2, all gene segments, node 1). The N5 gene segments of the H5N5 viruses from 2020 had a common ancestor that was estimated to have circulated between March 2019 and April 2020 (Table 1, N5, node 1) (Figure 2, N5), and a common ancestor with the closest related LPAI virus, A/mallard/Novosibirsk region/999K/2018(H12N5) that circulated between September 2017 and August 2018. The tMRCA for the PA segments of the German, Belgian, and Danish H5N5 viruses found in 2020 also was estimated to be between April 2019 and March 2020 (Table 1, PA, node 2) (Figure 2, PA), descending from the same ancestor as the closest relative LPAI A/green sandpiper/Kurgan/1050/2018(H3N8) between January 2017 and February 2018 (Table 1, PA, node 3). The closest ancestor to the NP segment of the Danish H5N5 circulated between February 2015 and June 2016 (Table 1, NP, node 2) (Figure 2, NP), and the Danish H5N5 virus contained a PB2 segment that was closely related to LPAI viruses from Novosibirsk, with a common ancestor that likely circulated between June 2018 and March 2019 (Table 1, PB2, node 2) (Figure 2, PB2). The maximum clade credibility trees for the remaining segments can be found in Supplementary Figure S2, and their estimated tMRCAs in Table 1.

Sequence features typically associated with clade 2.3.4.4 H5 viruses were identified in the Danish HPAI H5N8 and H5N5 viruses (Supplementary Table S2). In addition, one of the H5N8 viruses (A/barnacle goose/Denmark/14139-3/2020) had a PB2-M64T amino acid substitution that is highly conserved in human influenza A H1N1, H2N2, and H3N2 viruses [21,22]. Screening all HPAI H5 viruses detected between 2018 and 2020 (available in the EpiFlu^TM^ Database (www.gisaid.org) on 12 January 2020) identified this amino acid substitution in only one other HPAI virus, A/chicken/Netherlands/20017694-004/2020(H5N8) (EPI_ISL_641395). The PB2-M64T substitution was not present in A/Astrakhan/3212/2020(H5N8) (EPI_ISL_1038924) virus detected in workers at a poultry farm in Russia on 12 December 2020.

## 4. Discussion

Starting from October 2020, HPAI clade 2.3.4.4b H5N8 and H5N5 viruses have affected wild birds and commercial poultry in Denmark and the rest of Europe. Phylogenetic analyses of the initially detected Danish H5N8 viruses revealed that they clustered with contemporary “Iraqi-like” European H5N8 viruses detected in the autumn of 2020. This is in agreement with analyses of contemporary German, Dutch, and UK HPAI H5 viruses [23]. Furthermore, we found that the viruses were genetically distinct from the European HPAI H5N8 viruses found in the first semester of 2020 and in the winter of 2016/2017. Taking into account the timing of detecting similar viruses in Russia and Kazakhstan in the summer of 2020, this also suggests that the H5N8 viruses from the autumn of 2020 were a new introduction of a different variant of the HPAI H5N8 virus, rather than being the result of continued local circulation of the H5N8 viruses from previous introductions. These new viruses were probably introduced by birds migrating from breeding grounds in Russia to overwintering places in Europe.

Our analyses showed that the Danish H5N5 virus contained an “Iraqi-like” H5N8 backbone and had reassorted with LPAI viruses, likely gaining its PB2, PA, and NA gene segments from Russian LPAI viruses and its NP gene segment from Eurasian LPAI viruses. This virus contained a unique genome segment constellation compared to contemporary H5N5 viruses detected in Germany, Belgium, and Russia. Since the Danish H5N5 virus probably arose from multiple reassortments, we calculated tMRCA for the gene segments PB2, PA, HA, NA, and NP. As the HA and NA gene segments of the Danish, German, Belgian, and Russian H5N5 viruses were highly similar, we hypothesize that these viruses first arose by a reassortment event between HPAI H5N8 and descendants of the most closely related Russian LPAI viruses, leading to the Russian H5N5 genotype sometime between September 2017 and April 2020. A subsequent reassortment event then likely occurred, resulting in the German and Belgian H5N5 genotype gaining its PA gene segment. The Danish H5N5 genotype possibly arose through further, successive reassortments to adopt the LPAI PB2 and NP gene segments; however, the time and the exact origin of these gene segments cannot be pinpointed due to the lack of closer relatives. In general, estimating the tMRCA and determining the origin of HPAI viruses is limited by under-surveillance of AIVs, as in many cases, there are years between the virus of interest and their closest relatives. Narrowing this informational gap would mitigate the challenges in understanding the ongoing evolution and emergence of HPAI viruses. It would also be informative to analyze the antigenic differences of the various H5 genotypes. However, standard and homologous reference sera within clade 2.3.4.4b were not available. The genetic characterization revealed that one of the Danish H5N8 viruses contained a PB2-M64T substitution. Computational analyses have shown that a threonine in position 64 is highly conserved in human influenza viruses [21,22]. This indicates that the substitution may be related to adaptation to humans; however, to the best of our knowledge, the impact of this mutation has not been tested in vitro or in vivo, and such investigations were out of the frame of the present study. The effect of this substitution on pathogenicity and zoonotic potential of AIV is therefore unknown and warrants further studies to provide data on the in vivo virulence and zoonotic potential. The remaining Danish H5N8 and H5N5 viruses did not contain any other markers or risk signatures besides typical clade 2.3.4.4b H5 virus signatures.

The ongoing evolution and periodic transmission of HPAI viruses to mammalian hosts calls for continued and extensive surveillance of AIVs in wild birds and poultry. While the Danish viruses analyzed in this study did not contain any known risk signatures, these investigations are vital for implementing risk-mitigation strategies and management of outbreaks.

## Figures and Tables

**Table 1:** Estimated tMRCA on PB2, PB1, PA, H5, NP, N5, N8, MP, and NS, and 95% HPD intervals calculated using BEAST analysis. The posterior probabilities are of the branch before the node. Node numbers are depicted in Figure 2 and in Supplementary Figure S2. tMRCA, time to most recent common ancestor; PB2, polymerase basic protein 2; PB1, polymerase basic protein 1; PA, polymerase acidic protein; H5, hemagglutinin subtype H5; NP, nucleoprotein; MP, matrix protein; NS, non-structural protein; HPD, highest posterior density.

	Node	tMRCA	95% HPD Interval	Posterior Probability
**PB2**	1 2	November 2019 November 2018	August 2019–February 2020 June 2018–March 2019	0.9998 0.7021
**PB1**	1	August 2019	May 2019–November 2019	0.9932
**PA**	1 2 3	September 2019 October 2019 August 2017	May 2019–December 2019 April 2019–March 2020 January 2017–February 2018	0.9984 1.0000 0.9267
**HA**	1 2	December 2019 June 2019	September 2019–February 2020 April 2019–September 2019	0.9995 1.0000
**NP**	1 2	October 2019 November 2015	June 2019–January 2020 February 2015–June 2016	1.0000 1.0000
**N5**	1 2	October 2019 March 2018	March 2019–April 2020 September 2017–August 2018	1.0000 0.8811
**N8**	1	January 2020	November 2019–April 2020	0.9999
**MP**	1	June 2019	January 2019–November 2019	1.0000
**NS**	1	September 2019	May 2019–December 2019	0.8207

## Data Availability

Consensus sequences generated in this study were submitted to the GISAID EpiFlu^TM^ sequence database, and their corresponding accession numbers are listed in Supplementary Table S1.

## Supplementary Materials

The following are available online at https://www.mdpi.com/article/10.3390/v13050886/s1, Figure S1: Maximum likelihood trees, Figure S2: Maximum clade credibility trees, Table S1: Details of the Danish 2020 HPAI H5 clade 2.3.4.4b viruses sequenced in this study, Table S2: Risk signatures screened for in the Danish 2020 HPAI clade 2.3.4.4b H5N8 and H5N5 viruses sequenced in this study.
