# Supplementary material for: Novel Clade 2.3.4.4b Highly Pathogenic Avian Influenza A H5N8 and H5N5 Viruses in Denmark, 2020
Source: Viruses. 2021 May 11;13(5):886. doi: 10.3390/v13050886 (PMC8151437; doi:10.3390/v13050886)
Supplement: Supplementary file 1 [file viruses-13-00886-s001.zip › viruses-1208701-supplemenary for conversion.pdf]

Supplementary Materials

Supplementary Figures

Figure S1. Maximum likelihood trees. Maximum likelihood trees for all influenza A virus segments showing the phylogenetic relationship of novel Danish HPAI clade 2.3.4.4 H5 virus sequences (coloured) from the autumn of 2020. Green: H5N5 wild bird virus, Red: H5N8 virus from poultry, Blue: H5N8 viruses from wild birds. The branch to the root, A/Goose/Guangdong/1/96 (broken line), has been shortened for visualisation purposes. The scale bar depicts nucleotide substitutions per site. HPAI, highly pathogenic avian influenza; PB2, polymerase basic protein 2; PB1, polymerase basic protein 1; PA, polymerase acidic protein; HA, hemagglutinin; NP, nucleoprotein; NA, neuraminidase; MP, matrix protein; NS, nonstructural protein.

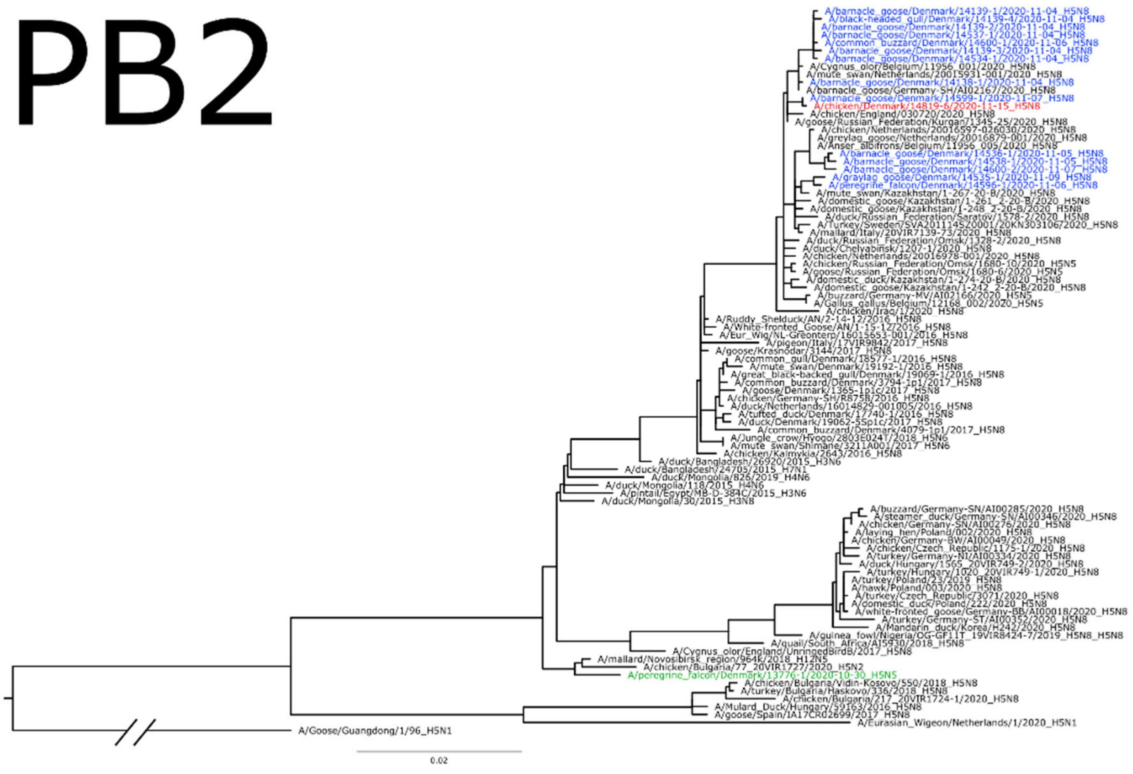

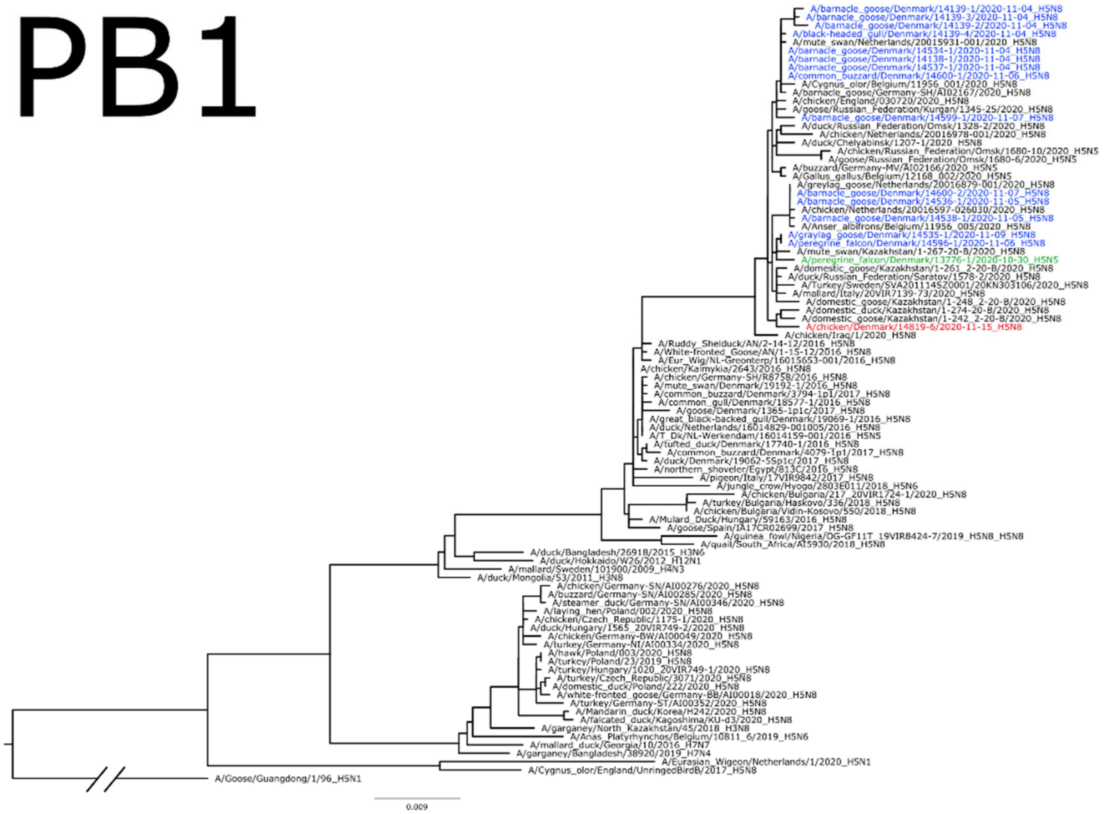
PB1
0.009

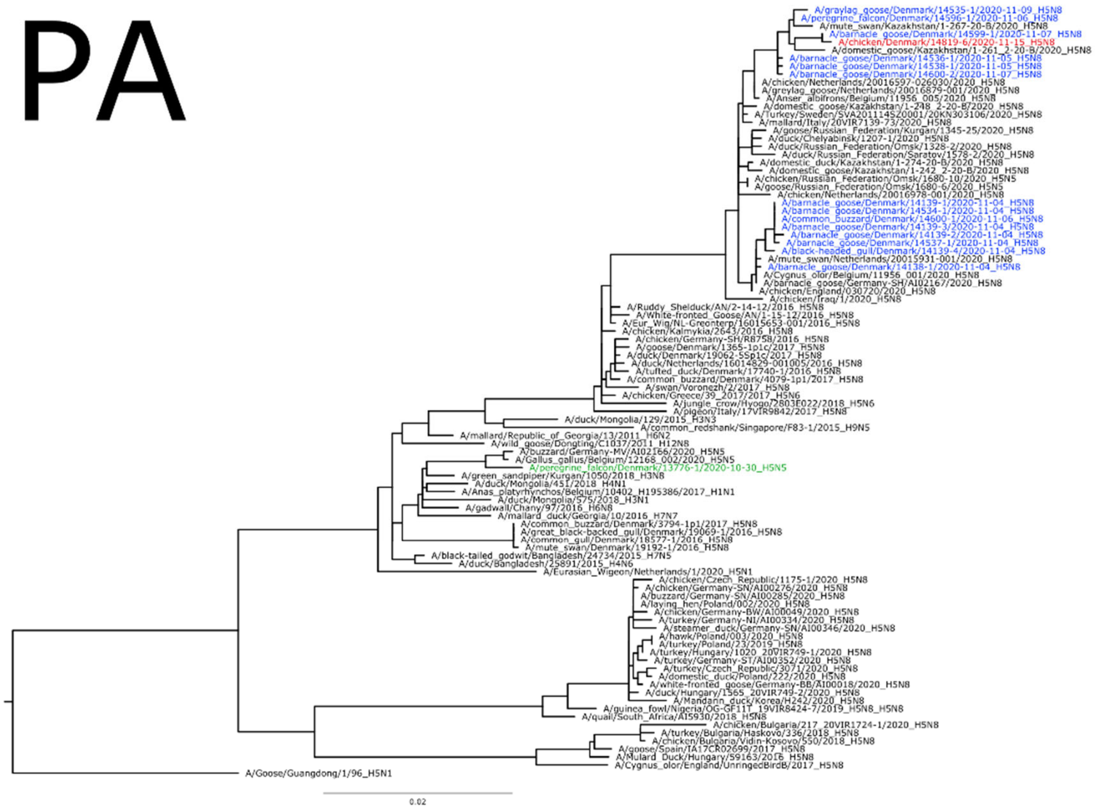
PA
0.02

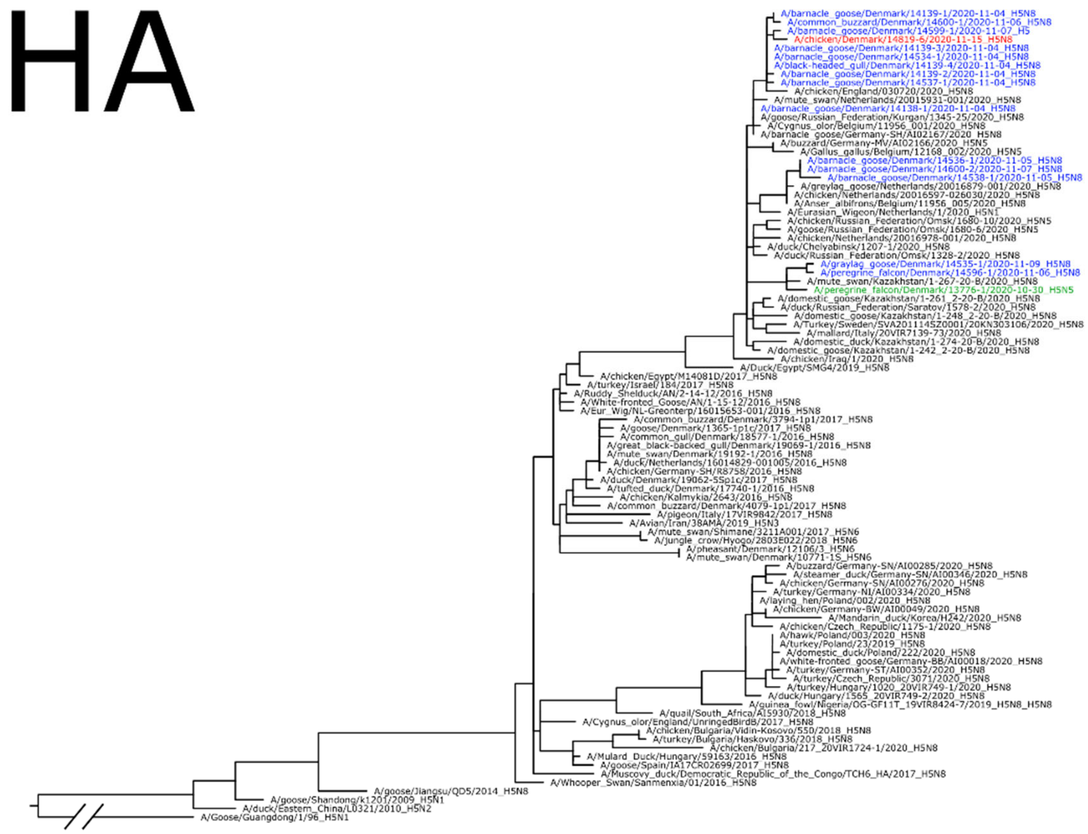
HA
A/barnacle_goose/Denmark/14139-1/2020-11-04_H5N8
A/common_buzzard/Denmark/14600-1/2020-11-06_H5N8
A/barnacle_goose/Denmark/14599-1/2020-11-07_H5
A/chicken/Denmark/14819-6/2020-11-15_H5N8
A/barnacle_goose/Denmark/14139-3/2020-11-04_H5N8
A/barnacle_goose/Denmark/14534-1/2020-11-04_H5N8
A/black-headed_gull/Denmark/14139-4/2020-11-04_H5N8
A/barnacle_goose/Denmark/14139-2/2020-11-04_H5N8
A/barnacle_goose/Denmark/14537-1/2020-11-04_H5N8
A/chicken/England/030720/2020_H5N8
A/mute_swan/Netherlands/20015931-001/2020_H5N8
A/barnacle_goose/Denmark/14138-1/2020-11-04_H5N8
A/goose/Russian_Federation/Kurgan/1345-25/2020_H5N8
A/Cygnus_olor/Belgium/11956_001/2020_H5N8
A/barnacle_goose/Germany-SH/AI02167/2020_H5N8
A/buzzard/Germany-MV/AI02166/2020_H5N5
A/Gallus_gallus/Belgium/12168_002/2020_H5N5
A/barnacle_goose/Denmark/14536-1/2020-11-05_H5N8
A/barnacle_goose/Denmark/14600-2/2020-11-07_H5N8
A/barnacle_goose/Denmark/14538-1/2020-11-05_H5N8
A/greylag_goose/Netherlands/20016879-001/2020_H5N8
A/chicken/Netherlands/20016597-026030/2020_H5N8
A/Anser_albifrons/Belgium/11956_005/2020_H5N8
A/Eurasian_Wigeon/Netherlands/1/2020_H5N1
A/chicken/Russian_Federation/Omsk/1680-10/2020_H5N5
A/goose/Russian_Federation/Omsk/1680-6/2020_H5N5
A/chicken/Netherlands/20016978-001/2020_H5N8
A/duck/Chelyabinsk/1207-1/2020_H5N8
A/duck/Russian_Federation/Omsk/1328-2/2020_H5N8
A/graylag_goose/Denmark/14535-1/2020-11-09_H5N8
A/peregrine_falcon/Denmark/14596-1/2020-11-06_H5N8
A/mute_swan/Kazakhstan/1-267-20-B/2020_H5N8
A/peregrine_falcon/Denmark/13776-1/2020-10-30_H5N5
A/domestic_goose/Kazakhstan/1-261_2-20-B/2020_H5N8
A/duck/Russian_Federation/Saratov/1578-2/2020_H5N8
A/domestic_goose/Kazakhstan/1-248_2-20-B/2020_H5N8
A/Turkey/Sweden/SVA201114SZ0001/20KN303106/2020_H5N8
A/mallard/Italy/20VIR7139-73/2020_H5N8
A/domestic_duck/Kazakhstan/1-274-20-B/2020_H5N8
A/domestic_goose/Kazakhstan/1-242_2-20-B/2020_H5N8
A/chicken/Iraq/1/2020_H5N8
A/Duck/Egypt/SMG4/2019_H5N8
A/chicken/Egypt/M14081D/2017_H5N8
A/turkey/Israel/184/2017_H5N8
A/Ruddy_Shelduck/AN/2-14-12/2016_H5N8
A/White-fronted_Goose/AN/1-15-12/2016_H5N8
A/Eur_Wig/NL-Greonterp/16015653-001/2016_H5N8
A/common_buzzard/Denmark/3794-1p1/2017_H5N8
A/goose/Denmark/1365-1p1c/2017_H5N8
A/common_gull/Denmark/18577-1/2016_H5N8
A/great_black-backed_gull/Denmark/19069-1/2016_H5N8
A/mute_swan/Denmark/19192-1/2016_H5N8
A/duck/Netherlands/16014829-001005/2016_H5N8
A/chicken/Germany-SH/R8758/2016_H5N8
A/duck/Denmark/19062-5Sp1c/2017_H5N8
A/tufted_duck/Denmark/17740-1/2016_H5N8
A/chicken/Kalmykia/2643/2016_H5N8
A/common_buzzard/Denmark/4079-1p1/2017_H5N8
A/pigeon/Italy/17VIR9842/2017_H5N8
A/Avian/Iran/38AMA/2019_H5N3
A/mute_swan/Shimane/3211A001/2017_H5N6
A/jungle_crow/Hyogo/2803E022/2018_H5N6
A/pheasant/Denmark/12106/3_H5N6
A/mute_swan/Denmark/10771-1S_H5N6
A/buzzard/Germany-SN/AI00285/2020_H5N8
A/steamer_duck/Germany-SN/AI00346/2020_H5N8
A/chicken/Germany-SN/AI00276/2020_H5N8
A/turkey/Germany-NI/AI00334/2020_H5N8
A/laying_hen/Poland/002/2020_H5N8
A/chicken/Germany-BW/AI00049/2020_H5N8
A/Mandarin_duck/Korea/H242/2020_H5N8
A/chicken/Czech_Republic/1175-1/2020_H5N8
A/hawk/Poland/003/2020_H5N8
A/turkey/Poland/23/2019_H5N8
A/domestic_duck/Poland/222/2020_H5N8
A/white-fronted_goose/Germany-BB/AI00018/2020_H5N8
A/turkey/Germany-ST/AI00352/2020_H5N8
A/turkey/Czech_Republic/3071/2020_H5N8
A/turkey/Hungary/1020_20VIR749-1/2020_H5N8
A/duck/Hungary/1565_20VIR749-2/2020_H5N8
A/guinea_fowl/Nigeria/OG-GF11T_19VIR8424-7/2019_H5N8_H5N8
A/quail/South_Africa/AI5930/2018_H5N8
A/Cygnus_olor/England/UnringedBirdB/2017_H5N8
A/chicken/Bulgaria/Vidin-Kosovo/550/2018_H5N8
A/turkey/Bulgaria/Haskovo/336/2018_H5N8
A/chicken/Bulgaria/217_20VIR1724-1/2020_H5N8
A/Mulard_Duck/Hungary/59163/2016_H5N8
A/goose/Spain/IA17CR02699/2017_H5N8
A/Muscovy_duck/Democratic_Republic_of_the_Congo/TCH6_HA/2017_H5N8
A/Whooper_Swan/Sanmenxia/01/2016_H5N8
A/goose/Jiangsu/QD5/2014_H5N8
A/goose/Shandong/k1201/2009_H5N1
A/duck/Eastern_China/L0321/2010_H5N2
A/Goose/Guangdong/1/96_H5N1

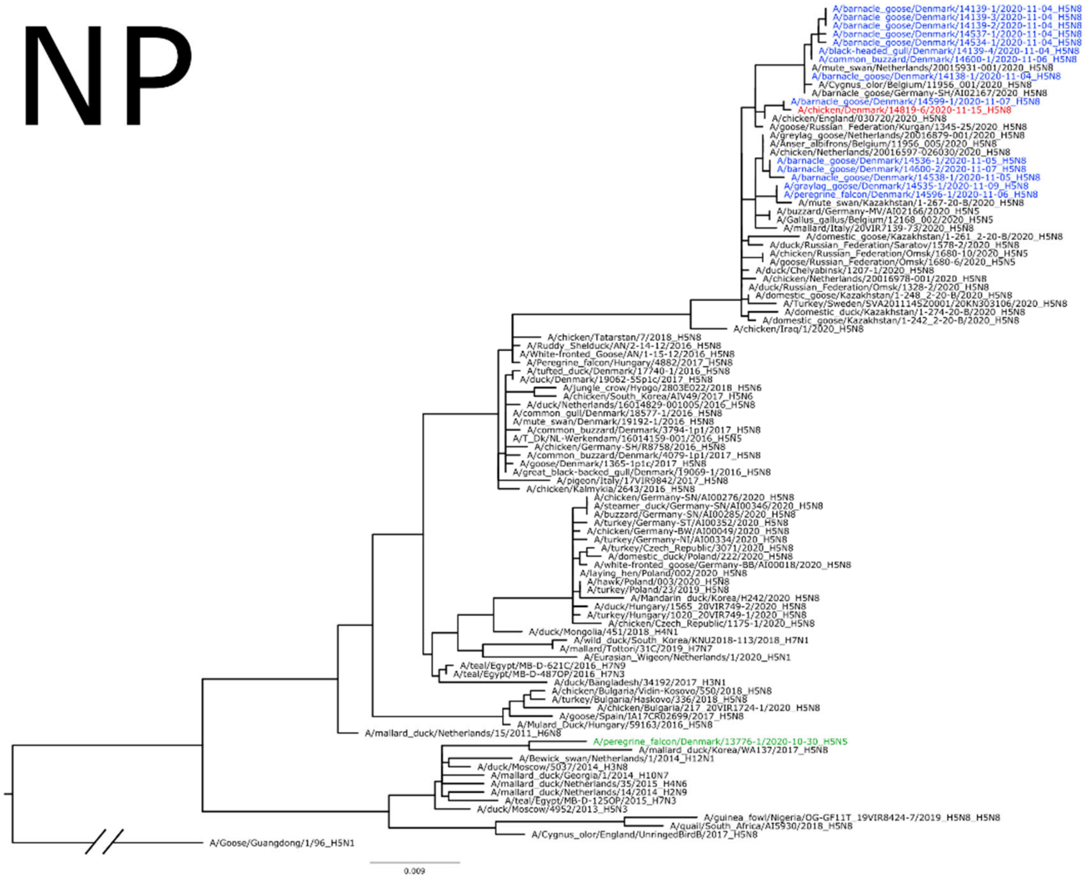
NP
0.009

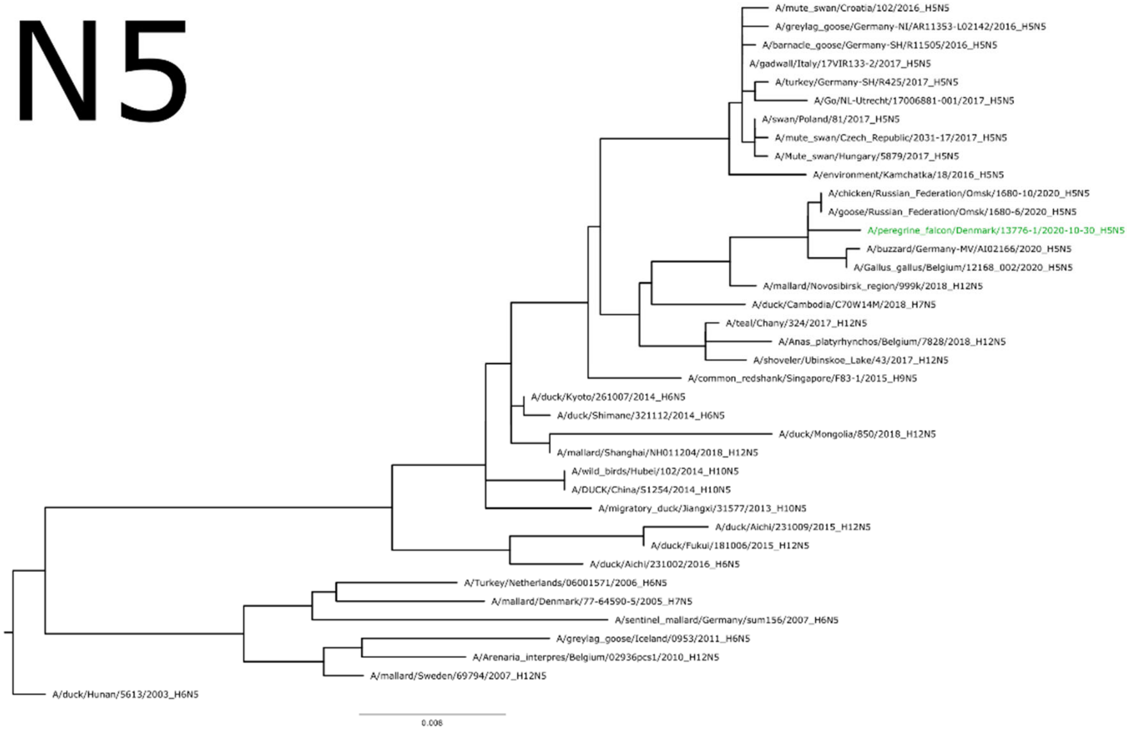
N5
A/mute_swan/Croatia/102/2016_H5N5
A/greylag_goose/Germany-NI/AR11353-L02142/2016_H5N5
A/barnacle_goose/Germany-SH/R11505/2016_H5N5
A/gadwall/Italy/17VIR133-2/2017_H5N5
A/turkey/Germany-SH/R425/2017_H5N5
A/Go/NL-Utrecht/17006881-001/2017_H5N5
A/swan/Poland/81/2017_H5N5
A/mute_swan/Czech_Republic/2031-17/2017_H5N5
A/Mute_swan/Hungary/5879/2017_H5N5
A/environment/Kamchatka/18/2016_H5N5
A/chicken/Russian_Federation/Omsk/1680-10/2020_H5N5
A/goose/Russian_Federation/Omsk/1680-6/2020_H5N5
A/peregrine_falcon/Denmark/13776-1/2020-10-30_H5N5
A/buzzard/Germany-MV/AI02166/2020_H5N5
A/Gallus_gallus/Belgium/12168_002/2020_H5N5
A/mallard/Novosibirsk_region/999k/2018_H12N5
A/duck/Cambodia/C70W14M/2018_H7N5
A/teal/Chany/324/2017_H12N5
A/Anas_platyrhynchos/Belgium/7828/2018_H12N5
A/shoveler/Ubinskoe_Lake/43/2017_H12N5
A/common_redshank/Singapore/F83-1/2015_H9N5
A/duck/Kyoto/261007/2014_H6N5
A/duck/Shimane/321112/2014_H6N5
A/duck/Mongolia/850/2018_H12N5
A/mallard/Shanghai/NH011204/2018_H12N5
A/wild_birds/Hubei/102/2014_H10N5
A/DUCK/China/S1254/2014_H10N5
A/migratory_duck/Jiangxi/31577/2013_H10N5
A/duck/Aichi/231009/2015_H12N5
A/duck/Fukui/181006/2015_H12N5
A/duck/Aichi/231002/2016_H6N5
A/Turkey/Netherlands/06001571/2006_H6N5
A/mallard/Denmark/77-64590-5/2005_H7N5
A/sentinel_mallard/Germany/sum156/2007_H6N5
A/greylag_goose/Iceland/0953/2011_H6N5
A/Arenaria_interpres/Belgium/02936pcs1/2010_H12N5
A/mallard/Sweden/69794/2007_H12N5
A/duck/Hunan/5613/2003_H6N5
0.008

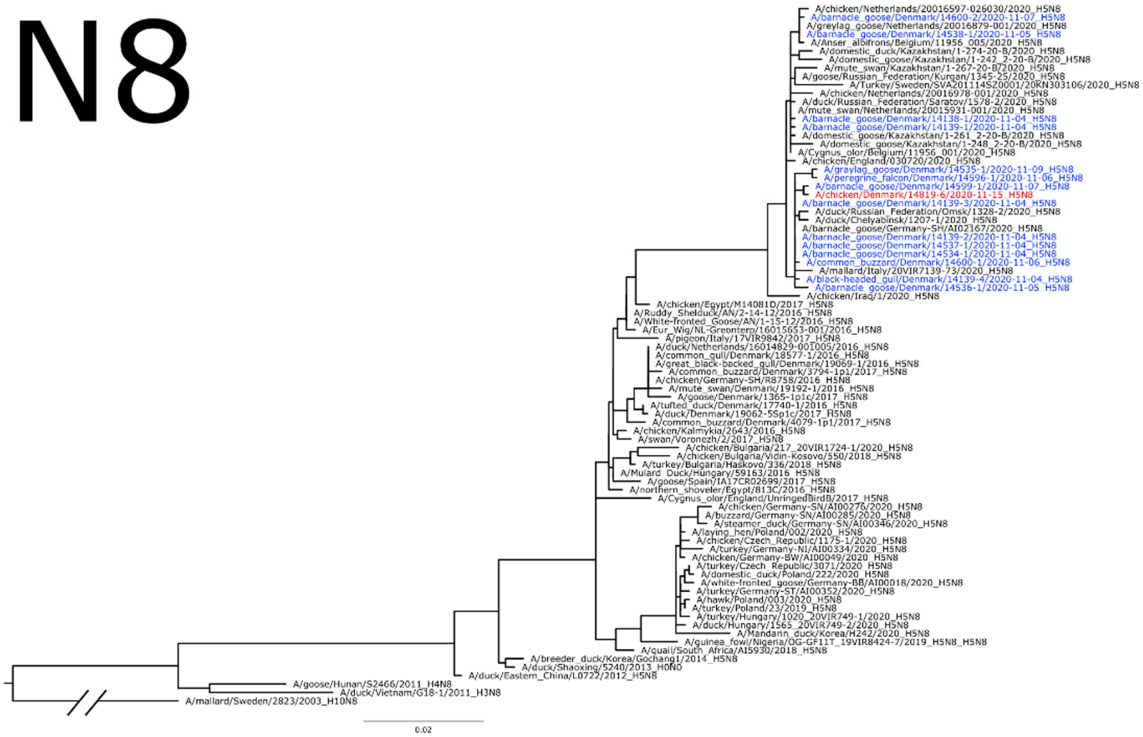
N8
0.02

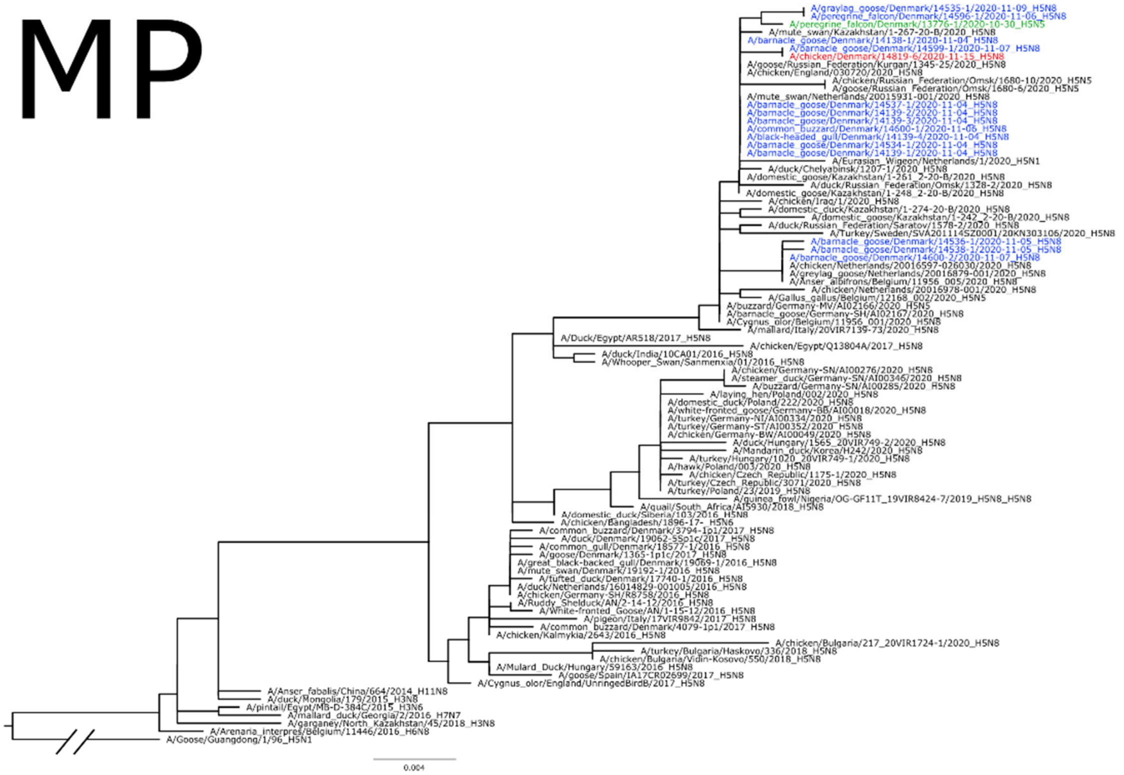
MP
0.004

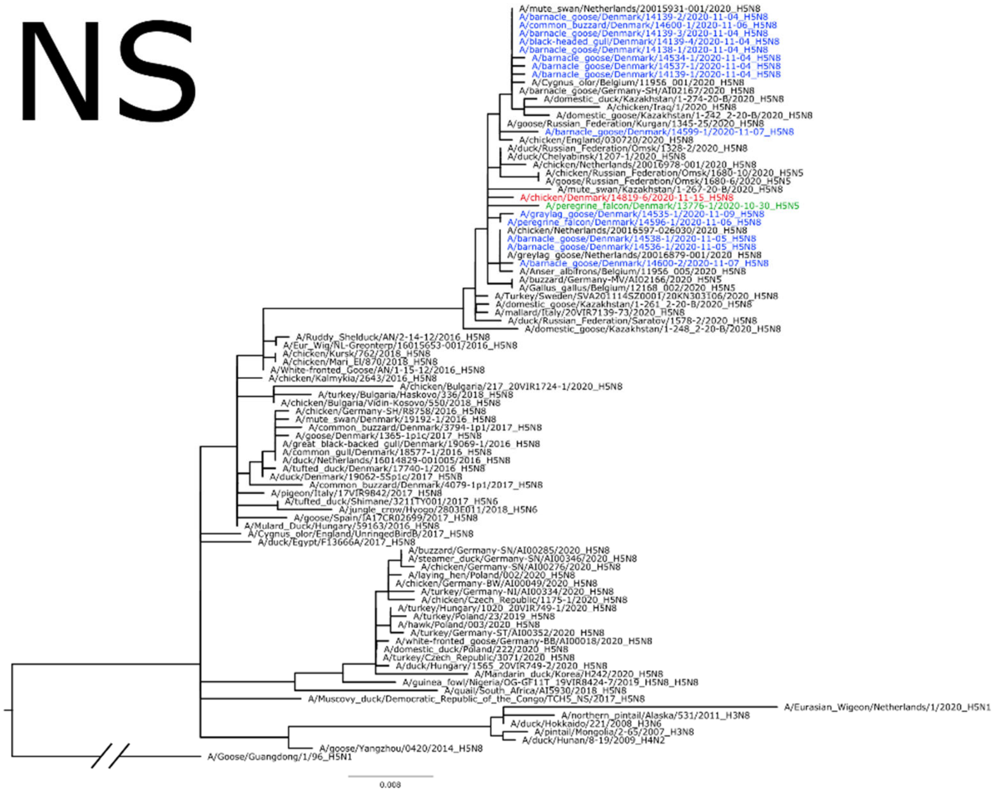
NS
A/mute_swan/Netherlands/20015931-001/2020_H5N8
A/barnacle_goose/Denmark/14139-2/2020-11-04_H5N8
A/common_buzzard/Denmark/14600-1/2020-11-06_H5N8
A/barnacle_goose/Denmark/14139-3/2020-11-04_H5N8
A/black-headed_gull/Denmark/14139-4/2020-11-04_H5N8
A/barnacle_goose/Denmark/14138-1/2020-11-04_H5N8
A/barnacle_goose/Denmark/14534-1/2020-11-04_H5N8
A/barnacle_goose/Denmark/14537-1/2020-11-04_H5N8
A/barnacle_goose/Denmark/14139-1/2020-11-04_H5N8
A/Cygnus_olor/Belgium/11956_001/2020_H5N8
A/barnacle_goose/Germany-SH/AI02167/2020_H5N8
A/domestic_duck/Kazakhstan/1-274-20-B/2020_H5N8
A/chicken/Iraq/1/2020_H5N8
A/domestic_goose/Kazakhstan/1-242_2-20-B/2020_H5N8
A/goose/Russian_Federation/Kurgan/1345-25/2020_H5N8
A/barnacle_goose/Denmark/14599-1/2020-11-07_H5N8
A/chicken/England/030720/2020_H5N8
A/duck/Russian_Federation/Omsk/1328-2/2020_H5N8
A/duck/Chelyabinsk/1207-1/2020_H5N8
A/chicken/Netherlands/20016978-001/2020_H5N8
A/chicken/Russian_Federation/Omsk/1680-10/2020_H5N5
A/goose/Russian_Federation/Omsk/1680-6/2020_H5N5
A/mute_swan/Kazakhstan/1-267-20-B/2020_H5N8
A/chicken/Denmark/14819-6/2020-11-15_H5N8
A/peregrine_falcon/Denmark/13776-1/2020-10-30_H5N5
A/graylag_goose/Denmark/14535-1/2020-11-09_H5N8
A/peregrine_falcon/Denmark/14596-1/2020-11-06_H5N8
A/chicken/Netherlands/20016597-026030/2020_H5N8
A/barnacle_goose/Denmark/14538-1/2020-11-05_H5N8
A/barnacle_goose/Denmark/14536-1/2020-11-05_H5N8
A/greylag_goose/Netherlands/20016879-001/2020_H5N8
A/barnacle_goose/Denmark/14600-2/2020-11-07_H5N8
A/Anser_albifrons/Belgium/11956_005/2020_H5N8
A/buzzard/Germany-MV/AI02166/2020_H5N5
A/Gallus_gallus/Belgium/12168_002/2020_H5N5
A/Turkey/Sweden/SVA201114SZ0001/20KN303106/2020_H5N8
A/domestic_goose/Kazakhstan/1-261_2-20-B/2020_H5N8
A/mallard/Italy/20VIR7139-73/2020_H5N8
A/duck/Russian_Federation/Saratov/1578-2/2020_H5N8
A/domestic_goose/Kazakhstan/1-248_2-20-B/2020_H5N8
A/Ruddy_Shelduck/AN/2-14-12/2016_H5N8
A/Eur_Wig/NL-Greonterp/16015653-001/2016_H5N8
A/chicken/Kursk/762/2018_H5N8
A/chicken/Mari_El/870/2018_H5N8
A/White-fronted_Goose/AN/1-15-12/2016_H5N8
A/chicken/Kalmykia/2643/2016_H5N8
A/chicken/Bulgaria/217_20VIR1724-1/2020_H5N8
A/turkey/Bulgaria/Haskovo/336/2018_H5N8
A/chicken/Bulgaria/Vidin-Kosovo/550/2018_H5N8
A/chicken/Germany-SH/R8758/2016_H5N8
A/mute_swan/Denmark/19192-1/2016_H5N8
A/common_buzzard/Denmark/3794-1p1/2017_H5N8
A/goose/Denmark/1365-1p1c/2017_H5N8
A/great_black-backed_gull/Denmark/19069-1/2016_H5N8
A/common_gull/Denmark/18577-1/2016_H5N8
A/duck/Netherlands/16014829-001005/2016_H5N8
A/tufted_duck/Denmark/17740-1/2016_H5N8
A/duck/Denmark/19062-5Sp1c/2017_H5N8
A/common_buzzard/Denmark/4079-1p1/2017_H5N8
A/pigeon/Italy/17VIR9842/2017_H5N8
A/tufted_duck/Shimane/3211TY001/2017_H5N6
A/jungle_crow/Hyogo/2803E011/2018_H5N6
A/goose/Spain/IA17CR02699/2017_H5N8
A/Mulard_Duck/Hungary/59163/2016_H5N8
A/Cygnus_olor/England/UnringedBirdB/2017_H5N8
A/duck/Egypt/F13666A/2017_H5N8
A/buzzard/Germany-SN/AI00285/2020_H5N8
A/steamer_duck/Germany-SN/AI00346/2020_H5N8
A/chicken/Germany-SN/AI00276/2020_H5N8
A/laying_hen/Poland/002/2020_H5N8
A/chicken/Germany-BW/AI00049/2020_H5N8
A/turkey/Germany-NI/AI00334/2020_H5N8
A/chicken/Czech_Republic/1175-1/2020_H5N8
A/turkey/Hungary/1020_20VIR749-1/2020_H5N8
A/turkey/Poland/23/2019_H5N8
A/hawk/Poland/003/2020_H5N8
A/turkey/Germany-ST/AI00352/2020_H5N8
A/white-fronted_goose/Germany-BB/AI00018/2020_H5N8
A/domestic_duck/Poland/222/2020_H5N8
A/turkey/Czech_Republic/3071/2020_H5N8
A/duck/Hungary/1565_20VIR749-2/2020_H5N8
A/Mandarin_duck/Korea/H242/2020_H5N8
A/guinea_fowl/Nigeria/OG-GF11T_19VIR8424-7/2019_H5N8_H5N8
A/quail/South_Africa/AI5930/2018_H5N8
A/Muscovy_duck/Democratic_Republic_of_the_Congo/TCH5_NS/2017_H5N8
A/Eurasian_Wigeon/Netherlands/1/2020_H5N1
A/northern_pintail/Alaska/531/2011_H3N8
A/duck/Hokkaido/221/2008_H3N6
A/pintail/Mongolia/2-65/2007_H3N8
A/duck/Hunan/8-19/2009_H4N2
A/goose/Yangzhou/0420/2014_H5N8
A/Goose/Guangdong/1/96_H5N1
0.008

Figure S2. Maximum Credibility Tree. Maximum credibility trees from strict molecular clock analyses of the segments PB1, N8, MP and NS of Danish HPAI clade 2.3.4.4 H5 virus sequences (colored) from the autumn of 2020. Green: H5N5 wild bird virus, Red: H5N8 virus from poultry, Blue: H5N8 viruses from wild birds. The scale bar depicts the timeline. PB1, polymerase basic protein 1; N8, neuraminidase subtype N8; MP, matrix protein; NS, nonstructural protein.

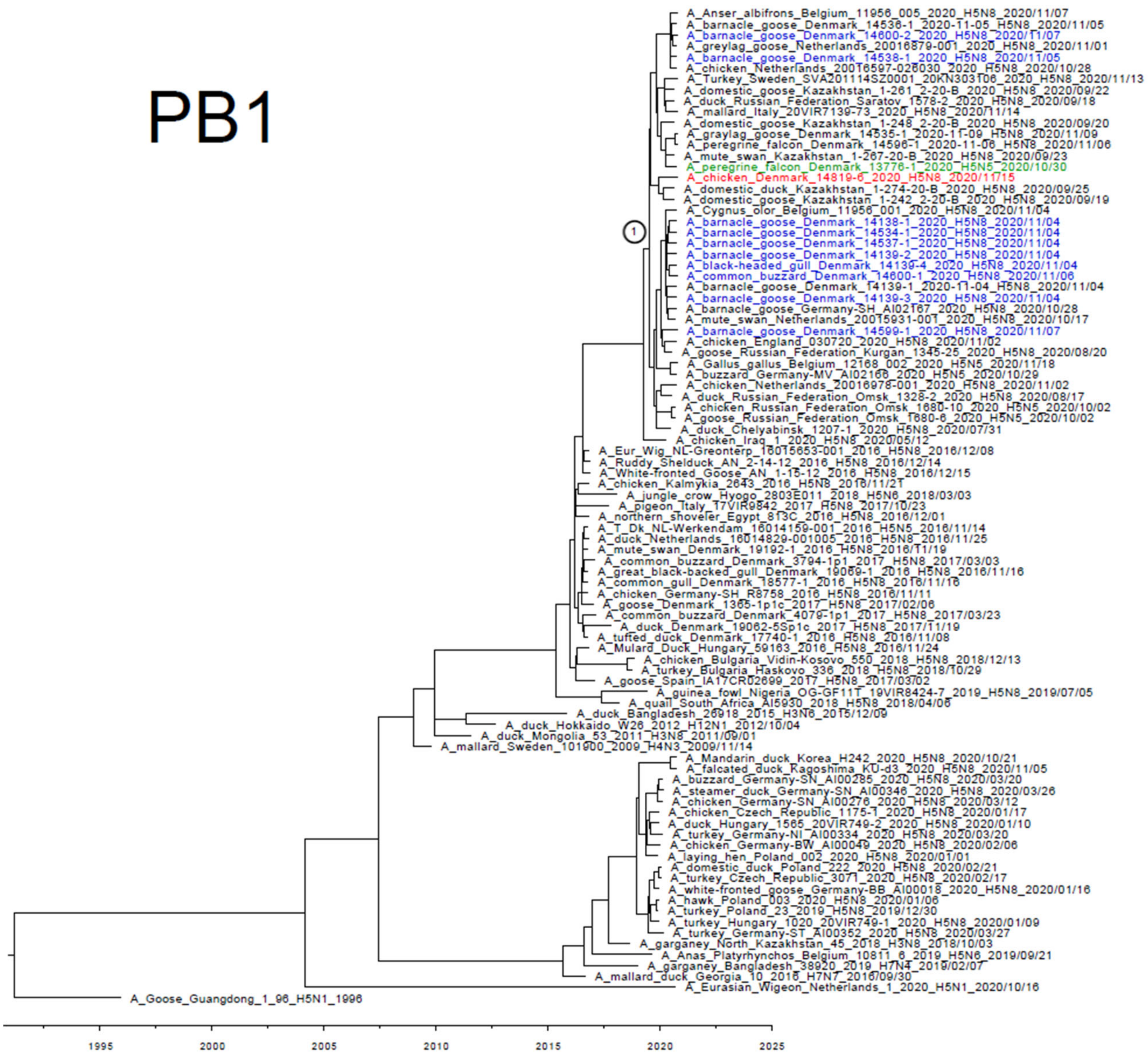

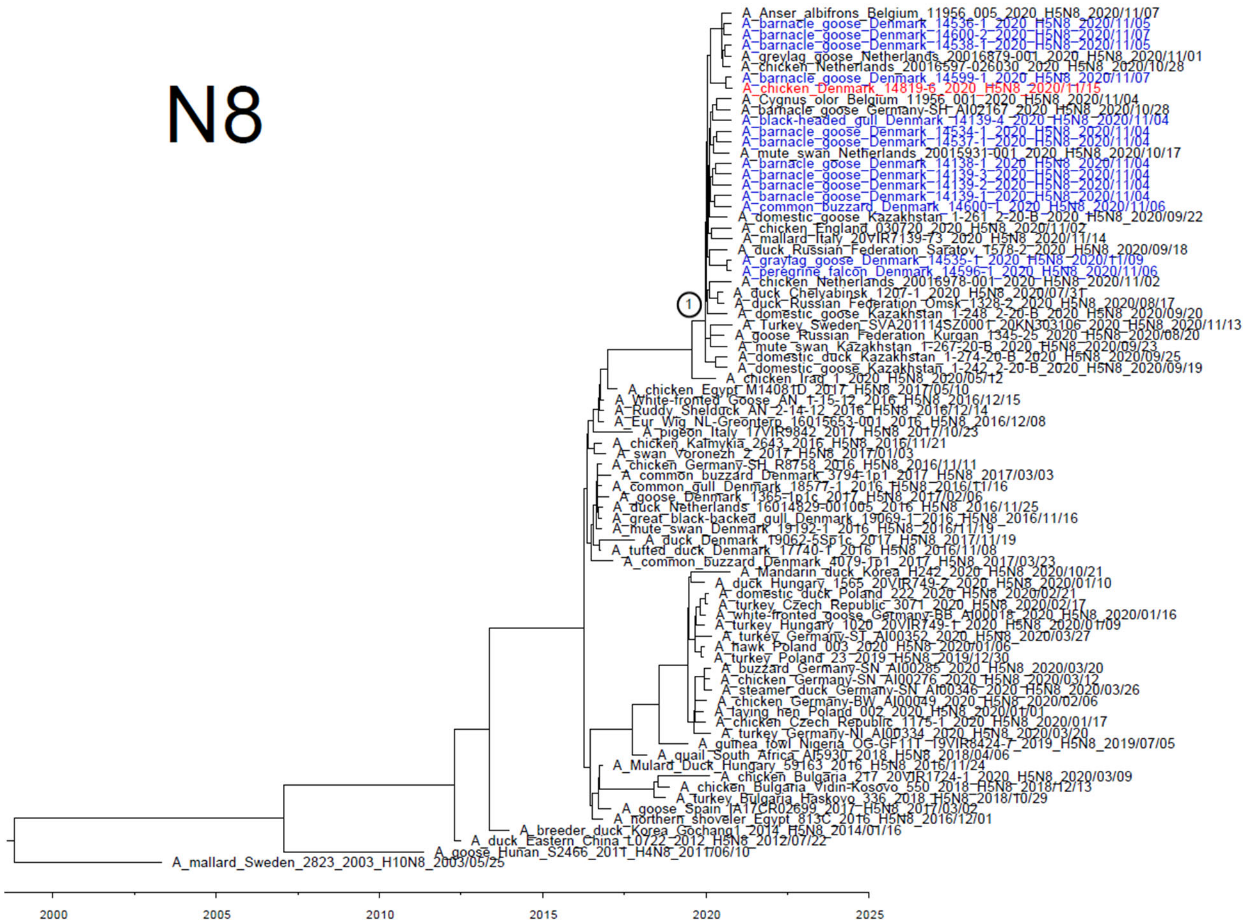
N8
A_Anser_albifrons_Belgium_11956_005_2020_H5N8_2020/11/07
A_barnacle_goose_Denmark_14536-1_2020_H5N8_2020/11/05
A_barnacle_goose_Denmark_14600-2_2020_H5N8_2020/11/07
A_barnacle_goose_Denmark_14538-1_2020_H5N8_2020/11/05
A_greylag_goose_Netherlands_20016879-001_2020_H5N8_2020/11/01
A_chicken_Netherlands_20016597-026030_2020_H5N8_2020/10/28
A_barnacle_goose_Denmark_14599-1_2020_H5N8_2020/11/07
A_chicken_Denmark_14819-6_2020_H5N8_2020/11/15
A_Cygnus_olor_Belgium_11956_001_2020_H5N8_2020/11/04
A_barnacle_goose_Germany-SH_AI02167_2020_H5N8_2020/10/28
A_black-headed_gull_Denmark_14139-4_2020_H5N8_2020/11/04
A_barnacle_goose_Denmark_14534-1_2020_H5N8_2020/11/04
A_barnacle_goose_Denmark_14537-1_2020_H5N8_2020/11/04
A_mute_swan_Netherlands_20015931-001_2020_H5N8_2020/10/17
A_barnacle_goose_Denmark_14138-1_2020_H5N8_2020/11/04
A_barnacle_goose_Denmark_14139-3_2020_H5N8_2020/11/04
A_barnacle_goose_Denmark_14139-2_2020_H5N8_2020/11/04
A_barnacle_goose_Denmark_14139-1_2020_H5N8_2020/11/04
A_common_buzzard_Denmark_14600-1_2020_H5N8_2020/11/06
A_domestic_goose_Kazakhstan_1-261_2-20-B_2020_H5N8_2020/09/22
A_chicken_England_030720_2020_H5N8_2020/11/02
A_mallard_Italy_20VIR7139-73_2020_H5N8_2020/11/14
A_duck_Russian_Federation_Saratov_1578-2_2020_H5N8_2020/09/18
A_graylag_goose_Denmark_14535-1_2020_H5N8_2020/11/09
A_peregrine_falcon_Denmark_14596-1_2020_H5N8_2020/11/06
A_chicken_Netherlands_20016978-001_2020_H5N8_2020/11/02
A_duck_Chelyabinsk_1207-1_2020_H5N8_2020/07/31
1
A_duck_Russian_Federation_Omsk_1328-2_2020_H5N8_2020/08/17
A_domestic_goose_Kazakhstan_1-248_2-20-B_2020_H5N8_2020/09/20
A_Turkey_Sweden_SVA201114SZ0001_20KN303106_2020_H5N8_2020/11/13
A_goose_Russian_Federation_Kurgan_1345-25_2020_H5N8_2020/08/20
A_mute_swan_Kazakhstan_1-267-20-B_2020_H5N8_2020/09/23
A_domestic_duck_Kazakhstan_1-274-20-B_2020_H5N8_2020/09/25
A_domestic_goose_Kazakhstan_1-242_2-20-B_2020_H5N8_2020/09/19
A_chicken_Iraq_1_2020_H5N8_2020/05/12
A_chicken_Egypt_M14081D_2017_H5N8_2017/05/10
A_White-fronted_Goose_AN_1-15-12_2016_H5N8_2016/12/15
A_Ruddy_Shelduck_AN_2-14-12_2016_H5N8_2016/12/14
A_Eur_Wig_NL-Greonterp_16015653-001_2016_H5N8_2016/12/08
A_pigeon_Italy_17VIR9842_2017_H5N8_2017/10/23
A_chicken_Kalmykia_2643_2016_H5N8_2016/11/21
A_swan_Voronezh_2_2017_H5N8_2017/01/03
A_chicken_Germany-SH_R8758_2016_H5N8_2016/11/11
A_common_buzzard_Denmark_3794-1p1_2017_H5N8_2017/03/03
A_common_gull_Denmark_18577-1_2016_H5N8_2016/11/16
A_goose_Denmark_1365-1p1c_2017_H5N8_2017/02/06
A_duck_Netherlands_16014829-001005_2016_H5N8_2016/11/25
A_great_black-backed_gull_Denmark_19069-1_2016_H5N8_2016/11/16
A_mute_swan_Denmark_19192-1_2016_H5N8_2016/11/19
A_duck_Denmark_19062-5Sp1c_2017_H5N8_2017/11/19
A_tufted_duck_Denmark_17740-1_2016_H5N8_2016/11/08
A_common_buzzard_Denmark_4079-1p1_2017_H5N8_2017/03/23
A_Mandarin_duck_Korea_H242_2020_H5N8_2020/10/21
A_duck_Hungary_1565_20VIR749-2_2020_H5N8_2020/01/10
A_domestic_duck_Poland_222_2020_H5N8_2020/02/21
A_turkey_Czech_Republic_3071_2020_H5N8_2020/02/17
A_white-fronted_goose_Germany-BB_AI00018_2020_H5N8_2020/01/16
A_turkey_Hungary_1020_20VIR749-1_2020_H5N8_2020/01/09
A_turkey_Germany-ST_AI00352_2020_H5N8_2020/03/27
A_hawk_Poland_003_2020_H5N8_2020/01/06
A_turkey_Poland_23_2019_H5N8_2019/12/30
A_buzzard_Germany-SN_AI00285_2020_H5N8_2020/03/20
A_chicken_Germany-SN_AI00276_2020_H5N8_2020/03/12
A_steamer_duck_Germany-SN_AI00346_2020_H5N8_2020/03/26
A_chicken_Germany-BW_AI00049_2020_H5N8_2020/02/06
A_laying_hen_Poland_002_2020_H5N8_2020/01/01
A_chicken_Czech_Republic_1175-1_2020_H5N8_2020/01/17
A_turkey_Germany-NI_AI00334_2020_H5N8_2020/03/20
A_guinea_fowl_Nigeria_OG-GF11T_19VIR8424-7_2019_H5N8_2019/07/05
A_quail_South_Africa_AI5930_2018_H5N8_2018/04/06
A_Mulard_Duck_Hungary_59163_2016_H5N8_2016/11/24
A_chicken_Bulgaria_217_20VIR1724-1_2020_H5N8_2020/03/09
A_chicken_Bulgaria_Vidin-Kosovo_550_2018_H5N8_2018/12/13
A_turkey_Bulgaria_Haskovo_336_2018_H5N8_2018/10/29
A_goose_Spain_IA17CR02699_2017_H5N8_2017/03/02
A_northern_shoveler_Egypt_813C_2016_H5N8_2016/12/01
A_breeder_duck_Korea_Gochang1_2014_H5N8_2014/01/16
A_duck_Eastern_China_L0722_2012_H5N8_2012/07/22
A_goose_Hunan_S2466_2011_H4N8_2011/06/10
A_mallard_Sweden_2823_2003_H10N8_2003/05/25
2000
2005
2010
2015
2020
2025

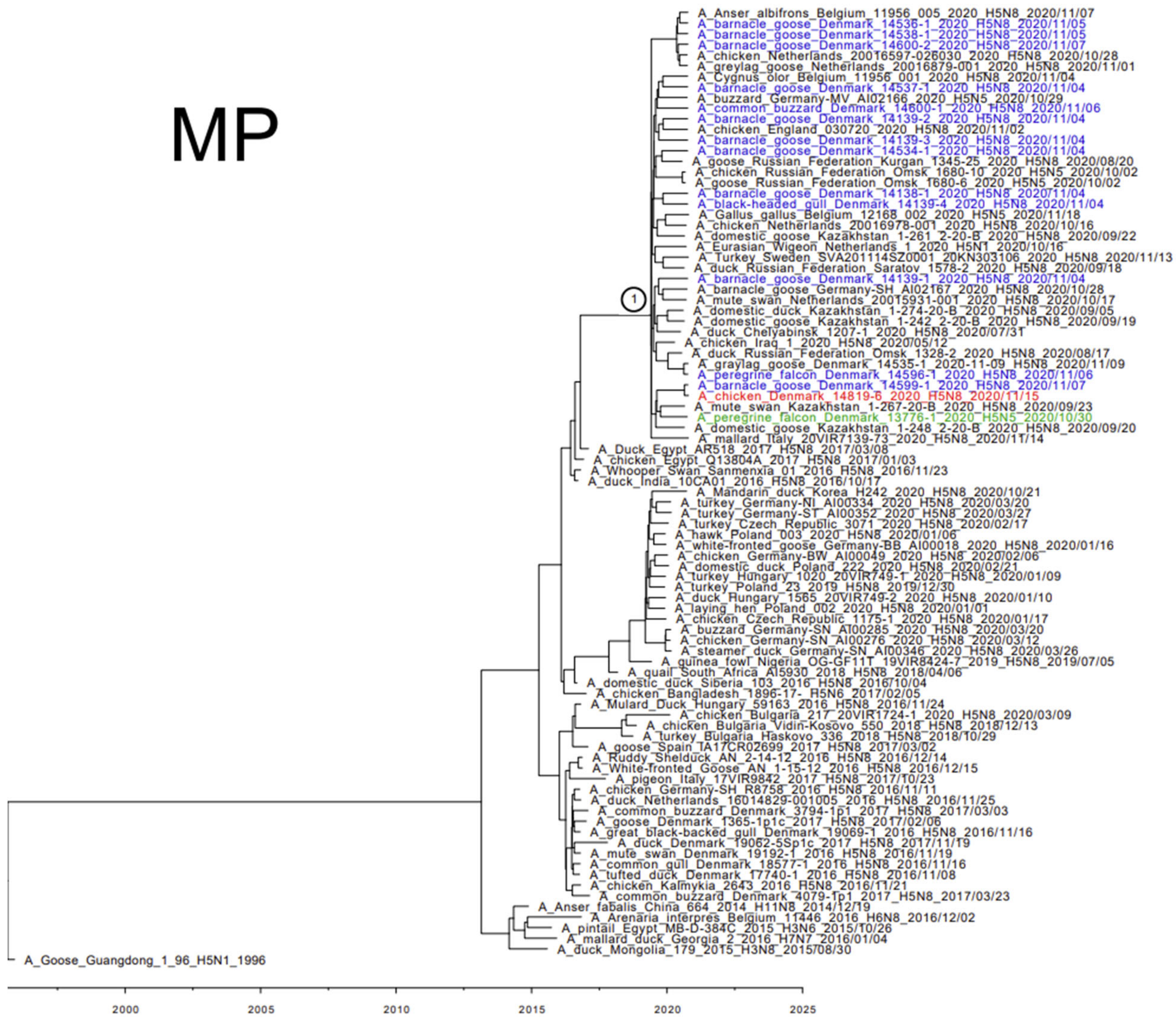
MP
A_Anser_albifrons_Belgium_11956_005_2020_H5N8_2020/11/07
A_barnacle_goose_Denmark_14536-1_2020_H5N8_2020/11/05
A_barnacle_goose_Denmark_14538-1_2020_H5N8_2020/11/05
A_barnacle_goose_Denmark_14600-2_2020_H5N8_2020/11/07
A_chicken_Netherlands_20016597-026030_2020_H5N8_2020/10/28
A_greylag_goose_Netherlands_20016879-001_2020_H5N8_2020/11/01
A_Cygnus_olor_Belgium_11956_001_2020_H5N8_2020/11/04
A_barnacle_goose_Denmark_14537-1_2020_H5N8_2020/11/04
A_buzzard_Germany-MV_AI02166_2020_H5N5_2020/10/29
A_common_buzzard_Denmark_14600-1_2020_H5N8_2020/11/06
A_barnacle_goose_Denmark_14139-2_2020_H5N8_2020/11/04
A_chicken_England_030720_2020_H5N8_2020/11/02
A_barnacle_goose_Denmark_14139-3_2020_H5N8_2020/11/04
A_barnacle_goose_Denmark_14534-1_2020_H5N8_2020/11/04
A_goose_Russian_Federation_Kurgan_1345-25_2020_H5N8_2020/08/20
A_chicken_Russian_Federation_Omsk_1680-10_2020_H5N5_2020/10/02
A_goose_Russian_Federation_Omsk_1680-6_2020_H5N5_2020/10/02
A_barnacle_goose_Denmark_14138-1_2020_H5N8_2020/11/04
A_black-headed_gull_Denmark_14139-4_2020_H5N8_2020/11/04
A_Gallus_gallus_Belgium_12168_002_2020_H5N5_2020/11/18
A_chicken_Netherlands_20016978-001_2020_H5N8_2020/10/16
A_domestic_goose_Kazakhstan_1-261_2-20-B_2020_H5N8_2020/09/22
A_Eurasian_Wigeon_Netherlands_1_2020_H5N1_2020/10/16
A_Turkey_Sweden_SVA201114SZ0001_20KN303106_2020_H5N8_2020/11/13
A_duck_Russian_Federation_Saratov_1578-2_2020_H5N8_2020/09/18
A_barnacle_goose_Denmark_14139-1_2020_H5N8_2020/11/04
A_barnacle_goose_Germany-SH_AI02167_2020_H5N8_2020/10/28
A_mute_swan_Netherlands_20015931-001_2020_H5N8_2020/10/17
A_domestic_duck_Kazakhstan_1-274-20-B_2020_H5N8_2020/09/05
A_domestic_goose_Kazakhstan_1-242_2-20-B_2020_H5N8_2020/09/19
A_duck_Chelyabinsk_1207-1_2020_H5N8_2020/07/31
A_chicken_Iraq_1_2020_H5N8_2020/05/12
A_duck_Russian_Federation_Omsk_1328-2_2020_H5N8_2020/08/17
A_graylag_goose_Denmark_14535-1_2020-11-09_H5N8_2020/11/09
A_peregrine_falcon_Denmark_14596-1_2020_H5N8_2020/11/06
A_barnacle_goose_Denmark_14599-1_2020_H5N8_2020/11/07
A_chicken_Denmark_14819-6_2020_H5N8_2020/11/15
A_mute_swan_Kazakhstan_1-267-20-B_2020_H5N8_2020/09/23
A_peregrine_falcon_Denmark_13776-1_2020_H5N5_2020/10/30
A_domestic_goose_Kazakhstan_1-248_2-20-B_2020_H5N8_2020/09/20
A_mallard_Italy_20VIR7139-73_2020_H5N8_2020/11/14
1
A_Duck_Egypt_AR518_2017_H5N8_2017/03/08
A_chicken_Egypt_Q13804A_2017_H5N8_2017/01/03
A_Whooper_Swan_Sanmenxia_01_2016_H5N8_2016/11/23
A_duck_India_10CA01_2016_H5N8_2016/10/17
A_Mandarin_duck_Korea_H242_2020_H5N8_2020/10/21
A_turkey_Germany-NI_AI00334_2020_H5N8_2020/03/20
A_turkey_Germany-ST_AI00352_2020_H5N8_2020/03/27
A_turkey_Czech_Republic_3071_2020_H5N8_2020/02/17
A_hawk_Poland_003_2020_H5N8_2020/01/06
A_white-fronted_goose_Germany-BB_AI00018_2020_H5N8_2020/01/16
A_chicken_Germany-BW_AI00049_2020_H5N8_2020/02/06
A_domestic_duck_Poland_222_2020_H5N8_2020/02/21
A_turkey_Hungary_1020_20VIR749-1_2020_H5N8_2020/01/09
A_turkey_Poland_23_2019_H5N8_2019/12/30
A_duck_Hungary_1565_20VIR749-2_2020_H5N8_2020/01/10
A_laying_hen_Poland_002_2020_H5N8_2020/01/01
A_chicken_Czech_Republic_1175-1_2020_H5N8_2020/01/17
A_buzzard_Germany-SN_AI00285_2020_H5N8_2020/03/20
A_chicken_Germany-SN_AI00276_2020_H5N8_2020/03/12
A_steamer_duck_Germany-SN_AI00346_2020_H5N8_2020/03/26
A_guinea_fowl_Nigeria_OG-GF11T_19VIR8424-7_2019_H5N8_2019/07/05
A_quail_South_Africa_AI5930_2018_H5N8_2018/04/06
A_domestic_duck_Siberia_103_2016_H5N8_2016/10/04
A_chicken_Bangladesh_1896-17-_H5N6_2017/02/05
A_Mulard_Duck_Hungary_59163_2016_H5N8_2016/11/24
A_chicken_Bulgaria_217_20VIR1724-1_2020_H5N8_2020/03/09
A_chicken_Bulgaria_Vidin-Kosovo_550_2018_H5N8_2018/12/13
A_turkey_Bulgaria_Haskovo_336_2018_H5N8_2018/10/29
A_goose_Spain_IA17CR02699_2017_H5N8_2017/03/02
A_Ruddy_Shelduck_AN_2-14-12_2016_H5N8_2016/12/14
A_White-fronted_Goose_AN_1-15-12_2016_H5N8_2016/12/15
A_pigeon_Italy_17VIR9842_2017_H5N8_2017/10/23
A_chicken_Germany-SH_R8758_2016_H5N8_2016/11/11
A_duck_Netherlands_16014829-001005_2016_H5N8_2016/11/25
A_common_buzzard_Denmark_3794-1p1_2017_H5N8_2017/03/03
A_goose_Denmark_1365-1p1c_2017_H5N8_2017/02/06
A_great_black-backed_gull_Denmark_19069-1_2016_H5N8_2016/11/16
A_duck_Denmark_19062-5Sp1c_2017_H5N8_2017/11/19
A_mute_swan_Denmark_19192-1_2016_H5N8_2016/11/19
A_common_gull_Denmark_18577-1_2016_H5N8_2016/11/16
A_tufted_duck_Denmark_17740-1_2016_H5N8_2016/11/08
A_chicken_Kalmykia_2643_2016_H5N8_2016/11/21
A_common_buzzard_Denmark_4079-1p1_2017_H5N8_2017/03/23
A_Anser_fabalis_China_664_2014_H11N8_2014/12/19
A_Arenaria_interpres_Belgium_11446_2016_H6N8_2016/12/02
A_pintail_Egypt_MB-D-384C_2015_H3N6_2015/10/26
A_mallard_duck_Georgia_2_2016_H7N7_2016/01/04
A_duck_Mongolia_179_2015_H3N8_2015/08/30
A_Goose_Guangdong_1_96_H5N1_1996
2000
2005
2010
2015
2020
2025

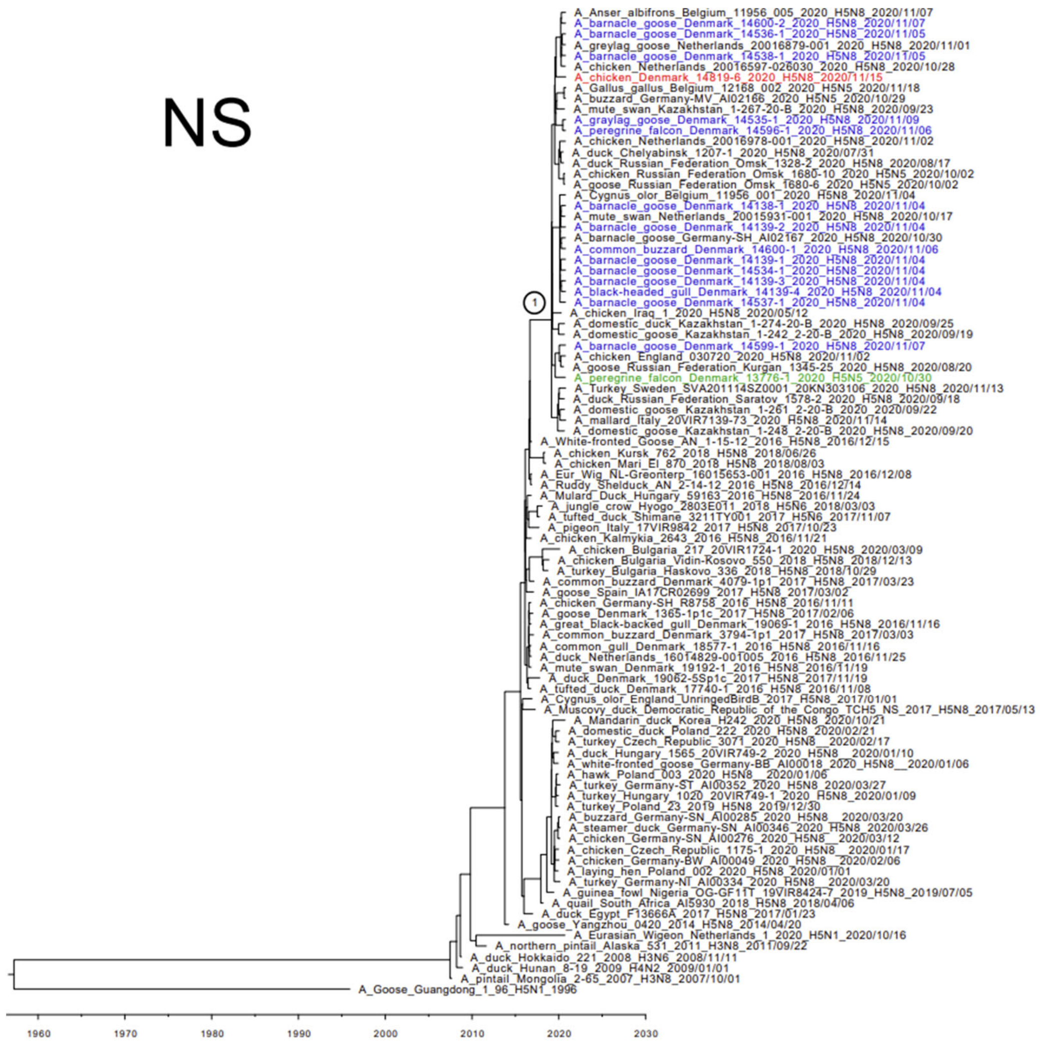
NS
A_Anser_albifrons_Belgium_11956_005_2020_H5N8_2020/11/07
A_barnacle_goose_Denmark_14600-2_2020_H5N8_2020/11/07
A_barnacle_goose_Denmark_14536-1_2020_H5N8_2020/11/05
A_greylag_goose_Netherlands_20016879-001_2020_H5N8_2020/11/01
A_barnacle_goose_Denmark_14538-1_2020_H5N8_2020/11/05
A_chicken_Netherlands_20016597-026030_2020_H5N8_2020/10/28
A_chicken_Denmark_14819-6_2020_H5N8_2020/11/15
A_Gallus_gallus_Belgium_12168_002_2020_H5N5_2020/11/18
A_buzzard_Germany-MV_AI02166_2020_H5N5_2020/10/29
A_mute_swan_Kazakhstan_1-267-20-B_2020_H5N8_2020/09/23
A_graylag_goose_Denmark_14535-1_2020_H5N8_2020/11/09
A_peregrine_falcon_Denmark_14596-1_2020_H5N8_2020/11/06
A_chicken_Netherlands_20016978-001_2020_H5N8_2020/11/02
A_duck_Chelyabinsk_1207-1_2020_H5N8_2020/07/31
A_duck_Russian_Federation_Omsk_1328-2_2020_H5N8_2020/08/17
A_chicken_Russian_Federation_Omsk_1680-10_2020_H5N5_2020/10/02
A_goose_Russian_Federation_Omsk_1680-6_2020_H5N5_2020/10/02
A_Cygnus_olor_Belgium_11956_001_2020_H5N8_2020/11/04
A_barnacle_goose_Denmark_14138-1_2020_H5N8_2020/11/04
A_mute_swan_Netherlands_20015931-001_2020_H5N8_2020/10/17
A_barnacle_goose_Denmark_14139-2_2020_H5N8_2020/11/04
A_barnacle_goose_Germany-SH_AI02167_2020_H5N8_2020/10/30
A_common_buzzard_Denmark_14600-1_2020_H5N8_2020/11/06
A_barnacle_goose_Denmark_14139-1_2020_H5N8_2020/11/04
A_barnacle_goose_Denmark_14534-1_2020_H5N8_2020/11/04
A_barnacle_goose_Denmark_14139-3_2020_H5N8_2020/11/04
A_black-headed_gull_Denmark_14139-4_2020_H5N8_2020/11/04
A_barnacle_goose_Denmark_14537-1_2020_H5N8_2020/11/04
1
A_chicken_Iraq_1_2020_H5N8_2020/05/12
A_domestic_duck_Kazakhstan_1-274-20-B_2020_H5N8_2020/09/25
A_domestic_goose_Kazakhstan_1-242_2-20-B_2020_H5N8_2020/09/19
A_barnacle_goose_Denmark_14599-1_2020_H5N8_2020/11/07
A_chicken_England_030720_2020_H5N8_2020/11/02
A_goose_Russian_Federation_Kurgan_1345-25_2020_H5N8_2020/08/20
A_peregrine_falcon_Denmark_13776-1_2020_H5N5_2020/10/30
A_Turkey_Sweden_SVA201114SZ0001_20KN303106_2020_H5N8_2020/11/13
A_duck_Russian_Federation_Saratov_1578-2_2020_H5N8_2020/09/18
A_domestic_goose_Kazakhstan_1-261_2-20-B_2020_2020/09/22
A_mallard_Italy_20VIR7139-73_2020_H5N8_2020/11/14
A_domestic_goose_Kazakhstan_1-248_2-20-B_2020_H5N8_2020/09/20
A_White-fronted_Goose_AN_1-15-12_2016_H5N8_2016/12/15
A_chicken_Kursk_762_2018_H5N8_2018/06/26
A_chicken_Mari_El_870_2018_H5N8_2018/08/03
A_Eur_Wig_NL-Greonterp_16015653-001_2016_H5N8_2016/12/08
A_Ruddy_Shelduck_AN_2-14-12_2016_H5N8_2016/12/14
A_Mulard_Duck_Hungary_59163_2016_H5N8_2016/11/24
A_jungle_crow_Hyogo_2803E011_2018_H5N6_2018/03/03
A_tufted_duck_Shimane_3211TY001_2017_H5N6_2017/11/07
A_pigeon_Italy_17VIR9842_2017_H5N8_2017/10/23
A_chicken_Kalmykia_2643_2016_H5N8_2016/11/21
A_chicken_Bulgaria_217_20VIR1724-1_2020_H5N8_2020/03/09
A_chicken_Bulgaria_Vidin-Kosovo_550_2018_H5N8_2018/12/13
A_turkey_Bulgaria_Haskovo_336_2018_H5N8_2018/10/29
A_common_buzzard_Denmark_4079-1p1_2017_H5N8_2017/03/23
A_goose_Spain_IA17CR02699_2017_H5N8_2017/03/02
A_chicken_Germany-SH_R8758_2016_H5N8_2016/11/11
A_goose_Denmark_1365-1p1c_2017_H5N8_2017/02/06
A_great_black-backed_gull_Denmark_19069-1_2016_H5N8_2016/11/16
A_common_buzzard_Denmark_3794-1p1_2017_H5N8_2017/03/03
A_common_gull_Denmark_18577-1_2016_H5N8_2016/11/16
A_duck_Netherlands_16014829-001005_2016_H5N8_2016/11/25
A_mute_swan_Denmark_19192-1_2016_H5N8_2016/11/19
A_duck_Denmark_19062-5Sp1c_2017_H5N8_2017/11/19
A_tufted_duck_Denmark_17740-1_2016_H5N8_2016/11/08
A_Cygnus_olor_England_UnringedBirdB_2017_H5N8_2017/01/01
A_Muscovy_duck_Democratic_Republic_of_the_Congo_TCH5_NS_2017_H5N8_2017/05/13
A_Mandarin_duck_Korea_H242_2020_H5N8_2020/10/21
A_domestic_duck_Poland_222_2020_H5N8_2020/02/21
A_turkey_Czech_Republic_3071_2020_H5N8__2020/02/17
A_duck_Hungary_1565_20VIR749-2_2020_H5N8__2020/01/10
A_white-fronted_goose_Germany-BB_AI00018_2020_H5N8__2020/01/06
A_hawk_Poland_003_2020_H5N8__2020/01/06
A_turkey_Germany-ST_AI00352_2020_H5N8_2020/03/27
A_turkey_Hungary_1020_20VIR749-1_2020_H5N8_2020/01/09
A_turkey_Poland_23_2019_H5N8_2019/12/30
A_buzzard_Germany-SN_AI00285_2020_H5N8__2020/03/20
A_steamer_duck_Germany-SN_AI00346_2020_H5N8_2020/03/26
A_chicken_Germany-SN_AI00276_2020_H5N8__2020/03/12
A_chicken_Czech_Republic_1175-1_2020_H5N8__2020/01/17
A_chicken_Germany-BW_AI00049_2020_H5N8__2020/02/06
A_laying_hen_Poland_002_2020_H5N8_2020/01/01
A_turkey_Germany-NI_AI00334_2020_H5N8__2020/03/20
A_guinea_fowl_Nigeria_OG-GF11T_19VIR8424-7_2019_H5N8_2019/07/05
A_quail_South_Africa_AI5930_2018_H5N8_2018/04/06
A_duck_Egypt_F13666A_2017_H5N8_2017/01/23
A_goose_Yangzhou_0420_2014_H5N8_2014/04/20
A_Eurasian_Wigeon_Netherlands_1_2020_H5N1_2020/10/16
A_northern_pintail_Alaska_531_2011_H3N8_2011/09/22
A_duck_Hokkaido_221_2008_H3N6_2008/11/11
A_duck_Hunan_8-19_2009_H4N2_2009/01/01
A_pintail_Mongolia_2-65_2007_H3N8_2007/10/01
A_Goose_Guangdong_1_96_H5N1_1996
1960
1970
1980
1990
2000
2010
2020
2030

Supplementary Tables

Table S1: Details of the Danish 2020 HPAI H5 clade 2.3.4.4b viruses sequenced in this study

Virus	Subtype	Sampling location in Denmark	Collection date	Material	Accession no.
A/peregrine falcon/Denmark/13776-1/2020	H5N5	Guldborgsund	30/10-2020	Pool of tracheal and cloacal swabs	EPI_ISL_644737
A/barnacle goose/Denmark/14138-1/2020	H5N8	Tønder	04/11-2020	Pool of tracheal and cloacal swabs	EPI_ISL_644824
A/barnacle goose/Denmark/14139-1/2020	H5N8	Tønder	04/11-2020	Pool of tracheal and cloacal swabs	EPI_ISL_984670
A/barnacle goose/Denmark/14139-2/2020	H5N8	Tønder	04/11-2020	Pool of tracheal and cloacal swabs	EPI_ISL_984673
A/barnacle goose/Denmark/14139-3/2020	H5N8	Tønder	04/11-2020	Pool of tracheal and cloacal swabs	EPI_ISL_984675
A/black-headed gull/Denmark/14139-4/2020	H5N8	Tønder	04/11-2020	Pool of tracheal and cloacal swabs	EPI_ISL_984680
A/barnacle goose/Denmark/14534-1/2020	H5N8	Tønder	04/11-2020	Pool of tracheal and cloacal swabs	EPI_ISL_984681
A/barnacle goose/Denmark/14537-1/2020	H5N8	Vejle	04/11-2020	Pool of tracheal and cloacal swabs	EPI_ISL_984684
A/barnacle goose/Denmark/14536-1/2020	H5N8	Aabenraa	05/11-2020	Pool of tracheal and cloacal swabs	EPI_ISL_984686
A/barnacle goose/Denmark/14538-1/2020	H5N8	Sønderborg	05/11-2020	Pool of tracheal and cloacal swabs	EPI_ISL_984688
A/peregrine falcon/Denmark/14596-1/2020	H5N8	Svendborg	06/11-2020	Pool of tracheal and cloacal swabs	EPI_ISL_984690
A/common buzzard/Denmark/14600-1/2020	H5N8	Aabenraa	06/11-2020	Pool of tracheal and cloacal swabs	EPI_ISL_984691
A/barnacle goose/Denmark/14600-2/2020	H5N8	Aabenraa	07/11-2020	Pool of tracheal and cloacal swabs	EPI_ISL_984692
A/barnacle goose/Denmark/14599-1/2020	H5N8	Kalundborg	07/11-2020	Pool of tracheal and cloacal swabs	EPI_ISL_984693
A/graylag goose/Denmark/14535-1/2020	H5N8	Aalborg	09/11-2020	Pool of tracheal and cloacal swabs	EPI_ISL_984694
A/chicken/Denmark/14819-6/2020†	H5N8	Randers	15/11-2020	Pool of five cloacal swabs	EPI_ISL_984695

†Virus from commercial poultry. Remaining viruses were from wild birds.

Table S2: Risk signatures screened for in the Danish 2020 HPAI clade 2.3.4.4b H5N8 and H5N5 viruses sequenced in this study

Protein	Position	Present	Phenotype	Reference
**PB2**	M64T	Yes†	Human adaptation	[1,2]
	Q591K	No	Virulence	[3]
	E627K	No	Virulence	[4]
	D701N	No	Mammalian adaptation	[5]
	S714R	No	Mammalian adaptation	[5]
**PB1**	V3A	No	Virulence	[6]
	H99Y	No	Airborne transmission	[7]
	N328K	No	Virulence	[6]
	I368V	No	Airborne transmission	[7]
	N375S	No	Virulence	[6]
**PA**	T85I	No	Mammalian adaptation	[8]
	T97I	No	Virulence and mammalian adaptation	[9]
	G186S	No	Mammalian adaptation	[8]
	L336M	No	Mammalian adaptation	[8]
	Q556R	No	Virulence and mammalian adaptation	[9]
	G631S	No	Virulence and mammalian adaptation	[10]
**HA**	D110N	No	Binding to a2,6	[11]
	H119Y, Y172A, Q238L, G240S	Only Y172A	Airborne transmission	[7]
	A149V	No	Virulence	[4]
**NP**	Q357K	No	Virulence	[12]
	K470R	No	Virulence	[13]
**NA**	Stalk deletion	No	Virulence	[14]
**M1**	N30D	Yes‡	Virulence	[15]
	Y215A	Yes‡	Virulence	[15]
**NS1**	P42S	Yes‡	Virulence	[16]
	D92E	No	Virulence	[17]
	F103L	Yes‡	Virulence	[18]
	M106I	Yes‡	Virulence	[18]
	deletion 80-84	No	Virulence	[17]

†Substitution only found in A/barnacle goose/Denmark/14139-3/2020. ‡Features of H5 clade 2.3.4.4b viruses.

Supplementary references

1. Finkelstein, D.B.; Mukatira, S.; Mehta, P.K.; Obenauer, J.C.; Su, X.; Webster, R.G.; Naeve, C.W. Persistent Host Markers in Pandemic and H5N1 Influenza Viruses. *J. Virol.* **2007**, *81*, 10292–10299, doi:10.1128/jvi.00921-07.

2. Wen, L.; Chu, H.; Wong, B.H.Y.; Wang, D.; Li, C.; Zhao, X.; Chiu, M.C.; Yuan, S.; Fan, Y.; Chen, H.; et al. Large-scale sequence analysis reveals novel human-adaptive markers in PB2 segment of seasonal influenza A viruses article. *Emerg. Microbes Infect.* **2018**, *7*, 1–12, doi:10.1038/s41426-018-0050-0.

3. Wang, C.; Lee, H.H.Y.; Yang, Z.F.; Mok, C.K.P.; Zhang, Z. PB2-Q591K mutation determines the pathogenicity of avian H9N2 influenza viruses for mammalian species. *PLoS One* **2016**, *11*, 1–16, doi:10.1371/journal.pone.0162163.

4. Wu, H.; Peng, X.; Lu, R.; Xu, L.; Liu, F.; Cheng, L.; Lu, X.; Yao, H.; Wu, N. Virulence of an H5N8 highly pathogenic avian influenza is enhanced by the amino acid substitutions PB2 E627K and HA A149V. *Infect. Genet. Evol.* **2017**, *54*, 347–354, doi:10.1016/j.meegid.2017.07.026.

5. Czudai-Matwich, V.; Otte, A.; Matrosovich, M.; Gabriel, G.; Klenk, H.-D. PB2 Mutations D701N and S714R Promote Adaptation of an Influenza H5N1 Virus to a Mammalian Host. *J. Virol.* **2014**, *88*, 8735–8742, doi:10.1128/jvi.00422-14.

6. Salomon, R.; Franks, J.; Govorkova, E.A.; Ilyushina, N.A.; Yen, H.L.; Hulse-Post, D.J.; Humberd, J.; Trichet, M.; Rehg, J.E.; Webby, R.J.; et al. The polymerase complex genes contribute to the high virulence of the human H5N1 influenza virus isolate

A/Vietnam/1203/04. *J. Exp. Med.* **2006**, *203*, 689–697, doi:10.1084/jem.20051938.

7. Herfst, S.; Schrauwen, E.J.A.; Linster, M.; Chutinimitkul, S.; de Wit, E.; Munster, V.J.; Sorrel, E.M.; Bestebroer, T.M.; Burke, D.F.; Smith, D.J.; et al. Airborne Transmission of Influenza A/H5N1 Virus Between Ferrets. *Science (80-.).* **2012**, *336*, 1534–1541, doi:10.1038/nature12476.

8. Bussey, K.A.; Desmet, E.A.; Mattiacio, J.L.; Hamilton, A.; Bradel-Tretheway, B.; Bussey, H.E.; Kim, B.; Dewhurst, S.; Takimoto, T. PA Residues in the 2009 H1N1 Pandemic Influenza Virus Enhance Avian Influenza Virus Polymerase Activity in Mammalian Cells. *J. Virol.* **2011**, *85*, 7020–7028, doi:10.1128/jvi.00522-11.

9. Choi, W.S.; Baek, Y.H.; Kwon, J.J.; Jeong, J.H.; Park, S.J.; Kim, Y. Il; Yoon, S.W.; Hwang, J.; Kim, M.H.; Kim, C.J.; et al. Rapid acquisition of polymorphic virulence markers during adaptation of highly pathogenic avian influenza H5N8 virus in the mouse. *Sci. Rep.* **2017**, *7*, 1–13, doi:10.1038/srep40667.

10. Hiromoto, Y.; Saito, T.; Lindstrom, S.; Nerome, K. Characterization of low virulent strains of highly pathogenic A/Hong Kong/156/97 (H5N1) virus in mice after passage in embryonated hens' eggs. *Virology* **2000**, *272*, 429–437, doi:10.1006/viro.2000.0371.

11. Su, Y.; Yang, H.Y.; Zhang, B.J.; Jia, H.L.; Tien, P. Analysis of a point mutation in H5N1 avian influenza virus hemagglutinin in relation to virus entry into live mammalian cells. *Arch. Virol.* **2008**, *153*, 2253–2261, doi:10.1007/s00705-008-0255-y.

12. Laleye, A.T.; Abolnik, C. Emergence of highly pathogenic H5N2 and H7N1 influenza A viruses from low pathogenic precursors by serial passage in ovo. *PLoS One* **2020**, *15*, 1–

18, doi:10.1371/journal.pone.0240290.

13. Chen, L.; Wang, C.; Luo, J.; Li, M.; Liu, H.; Zhao, N.; Huang, J.; Zhu, X.; Ma, G.; Yuan, G.; et al. Amino acid substitution K470R in the nucleoprotein increases the virulence of H5N1 influenza A virus in mammals. *Front. Microbiol.* **2017**, *8*, 1–12, doi:10.3389/fmicb.2017.01308.

14. Zhou, H.; Yu, Z.; Hu, Y.; Tu, J.; Zou, W.; Peng, Y.; Zhu, J.; Li, Y.; Zhang, A.; Yu, Z.; et al. The Special Neuraminidase Stalk-Motif Responsible for Increased Virulence and Pathogenesis of H5N1 Influenza A Virus. *PLoS One* **2009**, *4*, 1–8, doi:10.1371/journal.pone.0006277.

15. Fan, S.; Deng, G.; Song, J.; Tian, G.; Suo, Y.; Jiang, Y.; Guan, Y.; Bu, Z.; Kawaoka, Y.; Chen, H. Two amino acid residues in the matrix protein M1 contribute to the virulence difference of H5N1 avian influenza viruses in mice. *Virology* **2009**, *384*, 28–32, doi:10.1016/j.virol.2008.11.044.

16. Jiao, P.; Tian, G.; Li, Y.; Deng, G.; Jiang, Y.; Liu, C.; Liu, W.; Bu, Z.; Kawaoka, Y.; Chen, H. A Single-Amino-Acid Substitution in the NS1 Protein Changes the Pathogenicity of H5N1 Avian Influenza Viruses in Mice. *J. Virol.* **2008**, *82*, 1146–1154, doi:10.1128/jvi.01698-07.

17. Long, J.X.; Peng, D.X.; Liu, Y.L.; Wu, Y.T.; Liu, X.F. Virulence of H5N1 avian influenza virus enhanced by a 15-nucleotide deletion in the viral nonstructural gene. *Virus Genes* **2008**, *36*, 471–478, doi:10.1007/s11262-007-0187-8.

18. Dankar, S.K.; Wang, S.; Ping, J.; Forbes, N.E.; Keleta, L.; Li, Y.; Brown, E.G. Influenza

A virus NS1 gene mutations F103L and M106I increase replication and virulence. *Virol. J.* **2011**, *8*, 1–13, doi:10.1186/1743-422X-8-13.
